# Persistence to antihypertensive drug classes in uncomplicated hypertension: a nationwide Swedish cohort study

**DOI:** 10.1016/j.eclinm.2025.103696

**Published:** 2025-12-19

**Authors:** Karl Laurell, Stefan Gustafsson, Erik Lampa, Dave Zachariah, Sofia Ek, Karin Rådholm, Mats Martinell, Johan Sundström

**Affiliations:** a Department of Medical Sciences, Uppsala University, Sweden; b Department of Health, Medicine and Caring Sciences, Linköping University, Sweden; c Department of Public Health and Caring Sciences, Uppsala University, Sweden; d Department of Information Technology, Uppsala University, Sweden; e The George Institute for Global Health, University of New South Wales, Sydney, NSW, Australia

**Keywords:** Persistence, Medication adherence, Antihypertensive agents, Register study, Hypertension, First-line drug choice

## Abstract

**Background:**

Only half of patients appear adherent to antihypertensive treatment. If so, the effectiveness in prevention against adverse cardiovascular outcomes may depend more on a drugs’ side effects than its blood pressure effects. We hypothesized that initiating treatment with a particular antihypertensive drug class will determine later persistence.

**Methods:**

We applied a new-user trial emulation approach to multiple mandatory health-care registers in Sweden. All individuals starting antihypertensive treatment with an angiotensin receptor blocker (ARB), angiotensin converting enzyme inhibitor (ACEi), calcium channel blocker (CCB), thiazide/thiazide-like diuretic (TD), or single–pill combination of two drugs (SPC) in 2011–2018 were assessed for inclusion. Patients with prior cardiovascular disease or diabetes were excluded. Persistence was defined using dispensed prescription refills with at least 80% adherence. We applied multistate Poisson regression models adjusting for age, sex, obesity, birth country, education, income, marital status and year of initiation. The study period was January 1st 2011–December 31st 2019.

**Findings:**

A total of 341,182 patients were included. Initiating antihypertensive treatment with an ARB was associated with a larger proportion continuing their original drug class, or indeed any antihypertensive drug class, at any time during the five years of follow-up. At three years, 44.7% (CI 43.7–45.7) of the ARB initiators were continuously persistent to their original drug class. The corresponding number if intermittent discontinuations were allowed was 81.8% (CI 80.9–82.8). Overall, if intermittent discontinuations and changes between classes were allowed, about 80% of all new users of antihypertensive drugs (regardless of class at initiation) were persistent to at least one antihypertensive drug class over 5 years.

**Interpretation:**

Initiating treatment with an ARB is associated with the best long-term class persistence and least treatment changes. The “rule of halves” should be replaced with the insight that, after the first year, at least 4 out of 5 patients take their antihypertensive drug at least 4 out of 5 days.

**Funding:**

The study was funded by Anders Wiklöf, Region Uppsala Primary Health and Care, and the Geriatric Foundation, Uppsala Sweden. The computations were enabled by resources in project sens2020005 and sens2020598 provided by the Swedish National Infrastructure for Computing (SNIC) at UPPMAX, partially funded by the 10.13039/501100004359Swedish Research Council through grant agreement no. 2018-05973.

**Research in context**

**Evidence before this study**

Relevant articles were identified through PubMed searches from 1st of January 2004 to 15th of January 2025, by combining search words related to hypertension, persistence, adherence, drug classes, angiotensin receptor blockers (ARB) and calcium channel blockers. Systematic reviews and articles comparing persistence or adherence between the four antihypertensive drug classes recommended in the latest European guidelines (for treatment of uncomplicated hypertension) were included. The majority of studies indicated better persistence when treatment was initiated with an ARB but results were partly conflicting.

**Added value of this study**

We applied a new user trial emulation approach to all Swedish residents (≥40 years old) initiating antihypertensive treatment for uncomplicated hypertension between January 1st 2011 and 31st of December 2018. This was done in the setting of universal healthcare, using several mandatory healthcare registries with minimal loss to follow-up. We developed a new framework to evaluate persistence while allowing for temporary discontinuations, and noted that at least 80% were taking at least one antihypertensive drug class with at least 80% adherence, over five years. Initiating treatment with an ARB was strongly associated with better persistence than other initial drug choices.

**Implications of all the available evidence**

As the effects on clinical outcomes of antihypertensive drugs are mediated by their blood pressure-lowering effects, with little evidence of other class effects, initiating treatment with an ARB has the potential to improve cardiovascular outcomes and decrease healthcare expenditures. The new knowledge that most patients continue to use their antihypertensive treatment over time challenges the earlier rule of halves and gives us a new perspective on where to best put our resources to improve outcomes.

## Introduction

High blood pressure is the risk factor leading to the largest numbers of premature deaths in the world,^1^ but only half of patients prescribed antihypertensive therapy are reported to be adherent to it.^2^ This is true even though effective and cheap medicines are readily available.^3^

In most current hypertension guidelines, four classes of antihypertensive drug classes are given equal footing as first-line monotherapy choices for treatment of uncomplicated hypertension.^4^, ^5^, ^6^, ^7^ In clinical practice, the effectiveness in prevention against adverse cardiovascular outcomes may be more dependent on a drugs’ tolerability and adherence than any blood pressure-lowering effects. The currently recommended fixed-combination strategy does not solve that problem. Although clinically significant differences in the degree of persistence between the recommended drug classes have been proposed,^8^, ^9^, ^10^, ^11^, ^12^ definite evidence to guide clinical practice is lacking.

Ideally, that question should be answered using pragmatic clinical trial designs. Since such trials are unlikely to be performed, we aimed to emulate such a trial using observational data.^13^

We hypothesized that the choice of initial drug treatment for uncomplicated hypertension affects long-term treatment persistence. To investigate this hypothesis, we emulated a clinical trial randomizing persons with uncomplicated hypertension between the recommended initial antihypertensive monotherapies and single–pill combinations. This was done in a nationwide sample of new users of antihypertensive drugs in the setting of universal healthcare and mandatory databases of Sweden.

## Methods

### Study design

This was an observational cohort study using participants’ unique 12-digit personal identity numbers to link the following mandatory registers: The National Prescribed Drug Register,^14^ the National Patient Register,^15^ the National Cause of Death Register,^16^ and the Longitudinal Integrated Database for Health Insurance and Labor Market Studies (LISA) register.^17^ The construction of the database has been previously described.^18^

Using the trial emulation framework^13^ the theoretical non-blinded randomized controlled trial optimal to answer our research question was designed (Table S1). The theoretical optimal trial was then used as a template to develop the most effective corresponding observational study. The study was registered before start of the study, and can be found in the HMA-EMA Catalogue with EU PAS number EUPAS50008 available on https://catalogues.ema.europa.eu/node/3539/administrative-details.

### Ethics

The study was approved by the Swedish Ethical Review Authority, number 2020-01556. Informed consent was waived because the study utilized anonymized registry data.

### Participants

#### Inclusion criteria

**Fig. 1:**
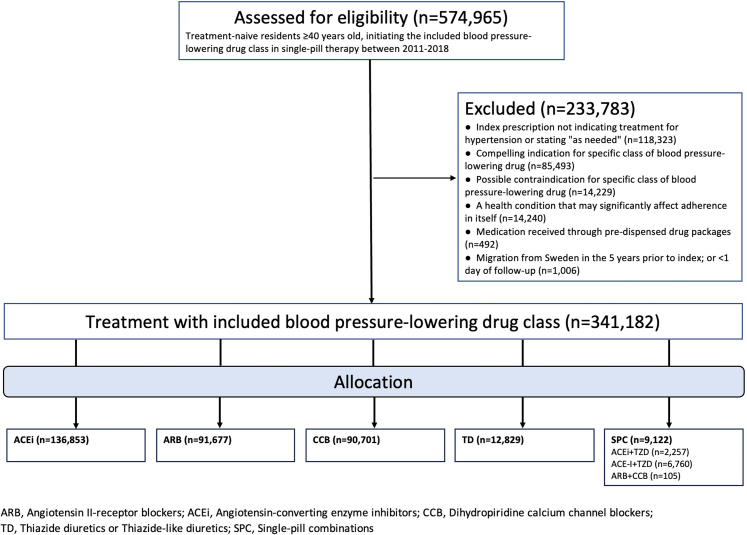
**Flow chart of included participants**.

Residents in Sweden ≥40 years old, receiving an antihypertensive drug for the first time and as a single pill between January 1st 2011 and the December 31st 2018 were identified (Fig. 1). Treatment naivety was ensured by retrospective evaluation of the five years prior to treatment start. If, during this time-period, an earlier collected prescription of any blood-pressure lowering drug (ATC code: C09, C07, C08, C03 and C02) was prevalent the individual was not included.

To comply with the latest ESC guidelines for the treatment of uncomplicated hypertension,^5^ only individuals initiating with an angiotensin receptor blocker (ARB), an angiotensin converting enzyme inhibitor (ACEi), a dihydropyridine calcium channel blocker (CCB), a thiazide/thiazide-like diuretic (TD) or a single–pill combination (SPC) of ARB + TD, ARB + CCB or ACEi + TD were included. No other SPCs have been used in Sweden.

During analysis, we noticed that (mainly) TD was sometimes prescribed as needed or for indications other than hypertension (mostly peripheral oedema). For this reason we chose to amend the inclusion criteria with the addition of index prescription needing to state the indication of hypertension, and excluding participants with their first prescription in the form of “as needed”.

#### Exclusion criteria

In order to emulate a clinical trial that could randomize persons to any of the recommended drug classes, we excluded persons who at baseline had a prevalent health condition that would strongly affect the exposure (the initial drug choice). These were conditions that presented a compelling indication or contraindication for a specific antihypertensive drug class, according to European guidelines contemporary with treatment initiation^19^ with the addition of stroke, peripheral oedema, hyponatremia and specified groups of medications that interact with one or more of the study medications (Tables S1 and S2).

We considered the following as compelling indications for one or more of the included drug classes: a diagnosis of heart failure, ischemic heart disease, diabetes, chronic kidney disease (stage ≥3), renovascular hypertension, atrial fibrillation, peripheral artery disease, asymptomatic atherosclerosis, aortic aneurysm, left ventricular hypertrophy (LVH), stroke and peripheral oedema; and the following as possible contraindications: gout, hyperaldosteronism, angioneurotic oedema, hyperkalemia, hypokalemia, hyponatremia and medications that interact with one or more of the included drug classes. Exclusion criteria (indications and contraindications) were identified using the National Patient Register (ICD-10 codes and registered medical and surgical procedures in secondary and tertiary care), information in the index prescription and by identifying concurrent prescriptions of medications indicative of the above diagnoses in the National Prescribed Drug Register.

We also excluded persons, who at baseline, had conditions that might strongly affect the outcome (medication persistence), including an ICD code of dementia, schizophrenia and some liver disorders (including alcohol mediated hepatic disease) on or at any time before baseline, or an ICD code of a malignant neoplasm, thyrotoxicosis/thyroiditis or secondary hypertension in the year before or on baseline. Lastly, patients retrieving their medication through predispensed drug packages, patients who during the 5 year period before baseline migrated back and forth to Sweden, and patients who had less than one day of follow up were excluded.

A complete list of inclusion and exclusion criteria can be found in Tables S1 and S2.

### Procedures

Information on all studied medications were collected together with the dosage information of all prescriptions from the national prescription register.^14^ The number of pills in each dispensation were divided by the dosage information of the corresponding prescription, providing a theoretical number of days that was covered by each dispensation. If the prescription contained unclear or no dosage information it was assumed to be 1 pill per day (the most common dosage). This was then compared to the subsequent number of days until a new dispensation or end of follow-up. The patient was defined as “on treatment” during time periods when she was retrieving dispensations covering at least 80% of the days (indicating ≥80% adherence). For example, if a patient retrieved a typical dispensation of 100 pills with dosage information 1 pill/day, this was assumed to last for 100/0.8 = 125 days.

If a new dispensation was retrieved before end of the nominal duration of the former dispensation with 100% adherence, the surplus was added to the following dispensations. At most, the total surplus was allowed to be 30 daily doses, and one year old. If a patient was indwelling at a hospital (according to the National Patient Register), the hospitalized days were added to the duration of the concurrent dispensation, since the patient would get her medication directly from the hospital.

Information regarding sex, income and other demographic information was retrieved from the LISA^17^ register and information regarding cause of death was retrieved from the National Cause of Death Register.^16^

### Outcomes

A patient that kept retrieving dispensations of the index drug class was defined as “class persistent” and a patient that kept retrieving dispensations of any of the studied antihypertensive drug classes was defined as “therapy persistent”. If a patient switched to another antihypertensive drug of the same class, or added other classes of antihypertensive drugs to the index class (as a free combination, or as a single–pill combination, SPC), she was still considered class persistent for as long as she kept retrieving refills containing the index class.

**Fig. 2:**
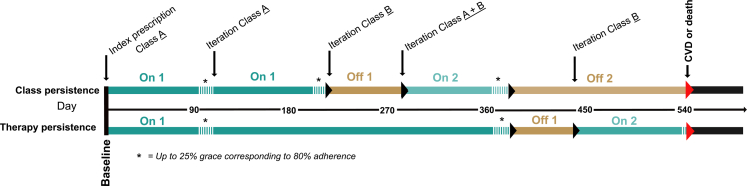
**Description of the different measures of persistence**. An individual retrieving refills of 90 pills each, with the dosage information of 1 pill per day of antihypertensive drug class A and B. The colors represent the individual moving between different on and off periods in the direction of the arrow. Class persistence: using the drug class prescribed at baseline. Therapy persistence: using any of the included antihypertensive drug classes. Continuous persistence: the proportion of individuals who are still in their first on period without in-between off periods (dark green). Examples: the individual in Fig. 2 is continuously class persistent until day 210 and continuously therapy persistent until day 380. Point persistence: the proportion of individuals who are in an on period at a chosen time point (any shade of green). Examples: the individual in Fig. 2 is class point persistent day 0–210 and 280–380 and therapy point persistent day 0–380 and 440–540. The colors and shades are the same as in Figs. 3 and 4.

To complement the definition of persistence used in earlier studies,^8^, ^9^, ^10^, ^11^^,^^20^, ^21^, ^22^ i.e. the proportion of participants continuously retrieving dispensations at specified timepoints since initiation (called “continuous persistence” in this study), we developed a novel aspect, that we call “point persistence”. Unlike the traditional definition, point persistence allows discontinuation periods and simply looks at the proportion on treatment, with at least 80% adherence, at a certain timepoint (according to a recently collected refill of one of the included antihypertensives). In addition, we followed how participants moved between on and off periods. The definitions are visualized in Fig. 2.

### Statistical analysis

Demographic data are presented as medians with interquartile range (IQR) or counts (%). Outcomes are presented as proportions, either observed proportions or estimated proportions with 95% confidence intervals (CI). Patients were censored when one of the following occurred: death, a cardiovascular event, emigration from Sweden or at the end of follow-up (31st of December 2019).

For the main analysis the cohort was divided into five strata: one for each class of monotherapy, and one aggregated for all the individuals starting treatment with an SPC.

The strata were analyzed in a multistate framework with transient states based on having more or less than 80% adherence to medication since last refill; hence, a patient could transition between being on or off treatment over time. Possible states and transitions were the following: on1 ⇒ off1 ⇒ on2⇒ off2 ⇒ on3 ⇒ off3 ⇒ on4 ⇒ off4 ⇒ on5+ ⇔ off5+, where the last transient states on5+ and off5+ corresponded to being on or off treatment on ≥5 occasions. The following diagnoses (ICD codes) were considered cardiovascular events: heart failure (I50, I11.0, I13), ischemic heart disease (I20-25), cerebrovascular disease (I60, I61, I62, I64, I63.0-I63.5, I63.8-I63.9, G45.0-3, G45.8-9) and renal failure ≥ stage 4 (N18.4-5). Together with all-cause death these cardiovascular events were treated as absorbing states.

An additional analysis was made within the SPC strata, but because of the limited number initiating treatment in each group, it could not be fit in our main model and only observational data are presented.

Causal assumptions were summarized using a direct acyclic graph (Figure S1), and used to develop bias-minimized models. In the multistate framework, Poisson regression models of the transition rates between all possible state transitions were fit as a function of time since treatment initiation, with adjustment for the following confounders: age, sex, obesity (ICD E66), birth country, education, income, marital status and the year for initiation of therapy, in separate models for each therapy type. Some continuous covariates were modelled using a thin plate spline as the smoothing basis and with k as the dimension of the basis including time since treatment initiation (k = 40), age (k = 15), and initiation year (k = 15). The levels of the categorical covariates are shown in Table 1. Based on the estimated transition rates, a binomial simulation was performed (as implemented in the simLexis function of the R package Epi) to generate the expected number and proportion of patients in given state at a given time for a given covariate combination (30,000 patients simulated with covariate combination). Percentile-based 95% confidence intervals were calculated from 1000 bootstrap draws.

**Table 1:** Sample characteristics.

	ACEi	ARB	CCB	TD	SPC
	(n = 136,853)	(n = 91,677)	(n = 90,701)	(n = 12,829)	(n = 9122)
Age, years	60.3 (52.2, 68.3)	60.2 (52.3, 68.3)	62.0 (53.2, 70.7)	65.3 (55.4, 74.2)	59.5 (51.8, 67.4)
Female, %	47.3	47.8	50.2	69.1	44.4
Diagnosis of obesity, %	1.6	1.6	1.5	1.6	1.6
Income, ∗100 SEK	2901 (1836, 3942)	3094 (1944, 4232)	2695 (1678, 3840)	2183 (1419, 3253)	2762 (1539, 3926)
Education, %					
Elementary	22.6	19.6	23.8	28.9	22.3
High School	47.9	47.2	46.7	44.2	44.7
Academic	27.5	30.9	27.2	24.9	26.9
Post graduate	1	1.1	1	0.7	1
Unknown	1	1.1	1.3	1.3	5.1
Civil status, %					
Partner	56	56.9	53.4	54.1	56.6
No partner	19.9	19.7	19.4	15.8	18.7
Separated	17.4	17	18.2	16.5	16.9
Widow	6.7	6.2	8.9	13.5	6.9
Unknown	0.1	0.2	0.1	0.1	0.9
Country of birth, %					
Sweden	82	84.6	79.0	78.2	73.9
Other Nordic countries	4.4	4.2	4.7	4.1	5.8
Europe outside the Nordic	5.5	4.6	5.6	4.6	8.3
North America	0.3	0.2	0.2	0.2	0.5
South America	0.6	0.5	0.5	0.4	0.6
Asia	3.6	3.3	3.9	2.4	7.4
Africa	0.9	0.7	1.1	0.8	1.3
Other	0.1	0.1	0.1	0.1	0.1
Unknown	2.6	1.9	4.8	9.3	2
Follow-up, years	5.3 (3.2, 7.3)	4.1 (2.5, 6.1)	4.3 (2.6, 6.5)	6.0 (3.8, 7.7)	4.8 (2.9, 6.7)
Index year	2014 (2012, 2016)	2015 (2013, 2017)	2015 (2013, 2017)	2013 (2012, 2015)	2015 (2013, 2016)

Data are % or median (IQR). ACEi, Angiotensin converting enzyme inhibitors; ARB, Angiotensin receptor blockers; CCB, dihydropyridine calcium channel blockers; TD, Thiazide or thiazide-like diuretic and SPC, single pill combinations.

The main results are presented as figures and point estimates (with confidence intervals) from the multistate model for the median male and female, i.e. corresponding to the covariate combination at baseline: time of initiation(2014-01-01), age(61 years), birth country(Sweden), highest education(high school), marital status(partner), total income(290,000 SEK), obesity(no) and sex(female or male). Further modelling was made for different values of the confounders (e.g. different ages) to test the results for generalizability to other covariate combinations.

A secondary analysis was performed without censoring for non-fatal cardiovascular events, to provide comparability to earlier studies.

To explore the possible effects of misclassification in the days covered by each dispensation, two sensitivity analyses were made: 1. Assuming a nominal duration of 100 days for every prescription (close to the median duration of the calculated days covered); 2. Assuming each dispensation had a nominal duration of 80 days per prescription (representing an extreme value).

Lastly, crude Kaplan–Meier estimates of time from treatment initiation to second and third dispensation were calculated for each medication class of the patients where the dispensations were calculated to last between 98 and 105 days (75% of the population). The statistical analysis was made using R (version # 4.3.1) with the following R packages: data.table, fst, survival, Epi, mgcv, parallel, ggplot2.

An additional non-parametric method using inverse probability weighting was also applied. It tested the robustness of our results, when the weights for the propensity score were altered to account for potential unmeasured confounding. The method evaluated the proportion of days adherent to treatment during the first year, and is described in the supplement.

### Role of funding source

The study was funded by Anders Wiklöf, Region Uppsala Primary Health and Care, and the Geriatric Foundation, Uppsala Sweden. The computations were enabled by resources in project sens2020005 and sens2020598 provided by the Swedish National Infrastructure for Computing (SNIC) at UPPMAX, partially funded by the Swedish Research Council through grant agreement no. 2018-05973. The funders had no role in study design; collection, analysis, and interpretation of data; writing of the report; or decision to submit the paper for publication.

## Results

Treatment-naive (n = 574,965) individuals that initiated regular treatment with antihypertensive monotherapy or SPC between January 1st 2011 and December 31st 2018 were assessed for eligibility. Of these, 233,783 (40.1%) were excluded because they fulfilled one or more of the exclusion criteria (Fig. 1) rendering a final cohort of 341,182 (median age 60.9 years, 48.8% women) patients available for analysis. Among these, the majority started treatment with an ACEi (40.1%) followed by ARB (26.9%), CCB (26.6%), TD (3.8%) and SPC (2.7%). Of the SPC users, 74.1% started with an ARB + TD, 24.7% with an ACEi + TD, and 1.2% with a ARB + CCB. Median duration of follow-up was 4.7 years, with a total of 1,636,607 person-years at risk. During the study, 28,428 individuals were censored because of death or a cardiovascular event. Characteristics of the sample are presented in Table 1.

One year after initiation of antihypertensive treatment, the observed proportion continuously persistent to the same drug class with adequate adherence and no interruptions between refills (continuous class persistence), was 68% for ARB, 56% for CCB, 55% for SPC *(ARB + TD = 57%, ARB + CCB = 52% and ACEi + TD = 40%)*, 53% for TD and 50% for ACEi. Observed proportions for other timepoints and persistence measures are presented in Figures S2 and S3.

**Fig. 3:**
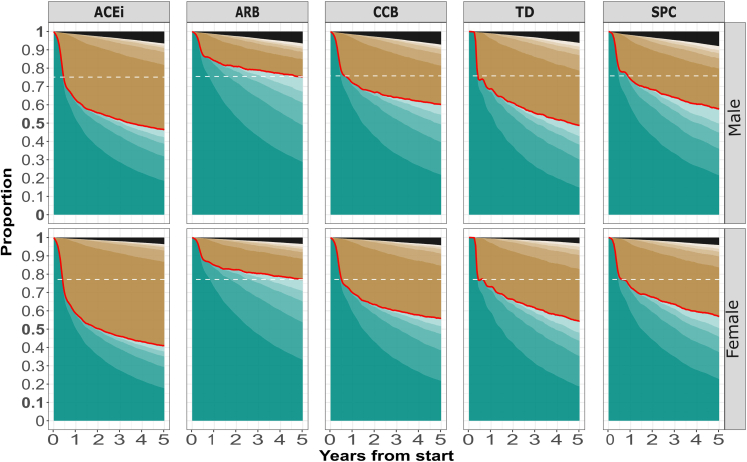
**Class persistence**. The different shades of green represents subsequent on periods and the different shades of brown represents subsequent off periods after initiation. Stronger colors represents earlier periods (e.g. the darkest green represents the first on period, the slightly lighter green represents the second on period etc). Black is an absorbing state representing death or a cardiovascular event. The darkest green (first on period) correspond to continuous persistence, while the lightest green correspond to point persistence. The dashed horizontal line is fitted to the class displaying the best persistence at the end of the time period. Data for the median male and female.

**Fig. 4:**
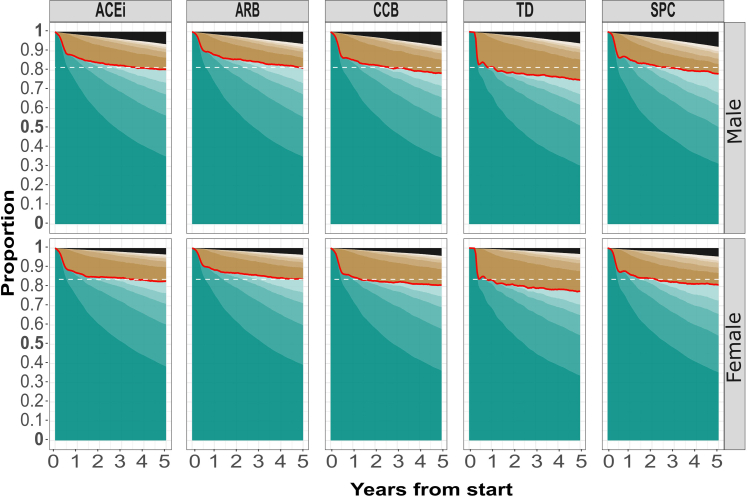
**Therapy persistence**. The different shades of green represents subsequent on periods and the different shades of brown represents subsequent off periods after initiation. Stronger colors represents earlier periods (e.g. the darkest green represents the first on period, the slightly lighter green represents the second on period etc.). Black is an absorbing state representing death or a cardiovascular event. The darkest green (first on period) correspond to continuous persistence, while the lightest green correspond to point persistence. The dashed horizontal line is fitted to the class displaying the best persistence at the end of the time period. Data for the median male and female.

Adjusting for age, sex, birth country, education, income, marital status and the year for initiation of therapy, men and women had similar persistence patterns (Figs. 3 and 4). Estimates of persistence are displayed in Tables 2 and 3 for the median male and female. In Figs. 3 and 4, an individual experiencing a cardiovascular event enters a black absorbing state, while in Tables 2 and 3 the same individual is censored.

**Table 2:** Class persistence at 1, 3 and 5 years.

Year	Sex	ACEi (95% CI)	ARB (95% CI)	CCB (95% CI)	TD (95% CI)	SPC (95% CI)
Continuous persistence						
1	M	53.0 (51.6–53.5)	71.1 (70.5–72.0)	60.4 (59.4–61.2)	55.2 (52.9–57.5)	59.8 (57.4–61.7)
	F	50.9 (49.8–51.7)	73.4 (72.7–74.2)	59.9 (59.1–60.9)	59.5 (57.5–61.3)	60.8 (58.2–62.7)
3	M	29.8 (28.6–30.6)	44.7 (43.7–45.7)	34.7 (33.8–35.8)	27.3 (24.9–29.5)	35.5 (32.5–37.5)
	F	27.9 (26.8–28.7)	47.8 (46.9–49.0)	34.1 (33.3–35.4)	32.5 (29.7–34.0)	36.3 (33.2–38.5)
5	M	19.7 (18.9–20.5)	31.0 (30.0–32.0)	23.3 (22.5–24.4)	16.1 (13.8–17.7)	23.7 (21.0–25.7)
	F	18.5 (17.2–18.9)	34.4 (33.3–35.4)	22.8 (22.0–24.0)	20.1 (17.9–21.6)	24.4 (21.6–26.5)
Point persistence						
1	M	63.4 (62.2–64.1)	85.3 (84.6–86.0)	73.2 (72.4–74.2)	69.6 (66.8–71.6)	75.3 (73.0–77.4)
	F	58.9 (57.7–59.6)	85.7 (84.8–86.4)	70.4 (69.6–71.4)	73.4 (71.1–75.3)	73.9 (71.0–75.8)
3	M	53.5 (52.6–54.9)	81.8 (80.9–82.8)	67.1 (66.0–68.4)	57.9 (54.3–60.8)	67.6 (64.3–70.3)
	F	46.5 (45.3–47.7)	81.6 (80.9–82.9)	61.9 (60.7–63.1)	62.7 (59.4–65.1)	64.9 (61.0–67.6)
5	M	49.5 (48.4–50.8)	80.2 (79.2–81.3)	64.5 (63.4–66.0)	52.1 (48.2–55.0)	62.8 (58.8–65.5)
	F	42.0 (40.4–42.9)	80.2 (78.8–81.0)	59.0 (57.4–60.0)	55.6 (53.1–59.3)	59.4 (54.8–62.1)

Data for the median male and female displayed as proportions in % with 95% confidence intervals. ACEi, Angiotensin converting enzyme inhibitors; ARB, Angiotensin receptor blockers; CCB, dihydropyridine calcium channel blockers; SPC, single pill combinations and TD, Thiazide- or thiazidlike diuretic. Individuals are censored from further analysis when and if death, CVD or end of follow up occurs.

**Table 3:** Therapy persistence at 1, 3 and 5 years.

Year	Sex	ACEi (95% CI)	ARB (95% CI)	CCB (95% CI)	TD (95% CI)	SPC (95% CI)
Continuous persistence						
1	M	73.4 (72.3–73.9)	75.2 (74.5–76.0)	72.5 (71.7–73.2)	67.8 (66.3–70.0)	69.2 (66.4–70.7)
	F	75.1 (74.0–75.6)	76.3 (76.2–77.6)	73.5 (72.7–74.2)	69.8 (68.3–71.7)	71.6 (68.9–72.9)
3	M	49.7 (48.6–50.7)	50.5 (49.5–51.6)	49.0 (48.1–50.1)	43.3 (41.8–46.7)	46.1 (42.6–48.1)
	F	52.6 (51.1–53.3)	52.9 (52.2–54.2)	50.6 (49.5–51.5)	46.4 (44.4–49.0)	49.3 (45.8–51.3)
5	M	37.4 (36.1–38.4)	37.4 (36.5–38.8)	36.7 (36.0–38.1)	31.7 (30.2–35.1)	34.1 (30.5–36.4)
	F	40.1 (38.8–41.1)	40.4 (39.3–41.5)	38.5 (37.4–39.5)	34.7 (32.8–37.5)	37.3 (33.7–39.3)
Point persistence						
1	M	87.7 (86.8–88.1)	89.3 (88.6–90.0)	86.2 (85.2–86.8)	82.3 (80.5–84.5)	87.1 (85.0–88.7)
	F	87.8 (86.7–88.1)	89.3 (88.9–90.1)	86.0 (84.9–86.4)	83.8 (82.0–85.4)	87.4 (85.1–88.8)
3	M	85.6 (85.0–86.5)	86.8 (86.5–88.1)	84.8 (83.6–85.4)	79.9 (77.7–82.6)	84.7 (82.6–86.9)
	F	86.0 (84.8–86.3)	87.1 (86.6–88.2)	84.1 (83.0–84.9)	80.6 (78.7–83.1)	84.5 (82.4–86.6)
5	M	86.0 (84.9–86.5)	86.7 (85.9–87.5)	84.6 (83.6–85.4)	79.6 (78.1–82.7)	84.7 (83.0–87.1)
	F	85.7 (84.8–86.3)	86.4 (85.8–87.5)	83.8 (82.8–84.8)	79.7 (78.5–82.9)	84.3 (82.5–86.9)

Data adjusted to the median male and female displayed as proportions in % with 95% confidence intervals. ACEi, Angiotensin converting enzyme inhibitors; ARB, Angiotensin receptor blockers; CCB, dihydropyridine calcium channel blockers; SPC, single pill combinations and TD, Thiazide- or thiazidlike diuretic. Individuals are censored from further analysis when and if death, CVD or end of follow up occurs. M, Male; F, Female.

For the monotherapy choices, continuous class persistence was highest for ARB followed by CCB for both sexes. ACEi had the lowest continuous class persistence for women at all timepoints but only at year one for men. Point class persistence displayed a similar trend but with ACEi being inferior to all other classes at all timepoints for both men and women.

Initiating treatment with an ARB was followed by higher therapy persistence compared to any of the other antihypertensive classes, both defined as continuous and point persistence. Of those that initiated treatment with ACEi, ARB or CCB, about 85% were using at least one antihypertensive drug with adequate adherence throughout follow-up and of those that started using TD, the corresponding figure was about 80%. In summary, at any timepoint during follow-up, at least 80% of the population are using at least one class of antihypertensive with at least 80% adherence.

Covariate combinations with different values for age, country of birth, obesity, educational level, marital status, income or year of initiation (Figures S9–S21) displayed similar hierarchies between the classes as in the main analysis. The same applied in models when participants were not censored, if (and when) a cardiovascular event occurred (Figures S22 and S23) and in models when each dispensation was assumed to cover 100 treatment days (Figures S26 and S27). Lastly, when results where modelled with each dispensation only covering 80 treatment days (Figures S24 and S25), ARB was still associated with best persistence but TD provided better class persistence than CCB.

The Kaplan–Meier analysis of time to retrieval of a second dispensation shows that a considerably larger proportion of ARB initiators collected a second dispensation of the same class compared to the other classes (Figure S5). Time to the third dispensation is displayed in Figure S6.

The additional non-parametric method, that accounts for potential unmeasured confounding and misspecification of treatment assignment models, produced results consistent with the one-year class persistence findings of the primary model (Figure S8). The proportion of days adherent to treatment during the first year remained consistently higher for ARB, further supporting the robustness of the primary study's conclusions.

## Discussion

In this nationwide trial emulation study, over 80% of persons that initiated antihypertensive treatment took at least one antihypertensive medication with at least 80% adherence over the five years after initiation. Initiating treatment with an ARB was strongly associated with a higher rate of continuation of the original drug class compared to initiation of any of the other drug class choices, implying lower healthcare utilization with an initial ARB choice.

The much higher adherence level in the present study than in previous studies is primarily explained by differences in methodology.^10^^,^^11^^,^^20^, ^21^, ^22^, ^23^, ^24^ In the present study we introduced a new metric, point persistence, which we believe reflects current patient-centered healthcare models better than the old metric, continuous persistence. With the advent of shared decision-making practices, the recognition of patients taking drug holidays, prioritizing lifestyle changes, temporarily forgetting their refills, or having other causes for not immediately or even continuously deciding on lifelong drug treatment, is a cornerstone of the building of therapeutic alliances. That said, the impact of temporary discontinuations on clinical outcomes needs more research.

Regarding differences in continuous persistence to the initial drug class, earlier results are somewhat conflicting,^10^^,^^20^^,^^22^ but most studies reported the best class persistence with ARB.^11^^,^^12^^,^^21^^,^^24^^,^^25^ Most of them included no or only a minority of patients initiating treatment after the expiry of the ARB patents (around 2010), making ARB a substantially more expensive choice in those studies, with high risk of selection bias. Later studies have also reported favorable class persistence with ARB.^8^^,^^23^^,^^24^ Lastly, an RCT comparing the four recommended antihypertensive drug classes head-to-head also observed best persistence with an ARB.^26^

A likely mechanism for the better persistence with ARB is fewer side effects than other drug classes; discontinuations due to adverse events have been on par with placebo for ARB but not for the other classes.^27^ The importance of adverse effects for discontinuation cannot be overstated; two out of three discontinuations have been proposed to be attributable to adverse effects.^26^

The lower persistence to ACEi in this study compared to some earlier studies^11^^,^^12^^,^^21^^,^^25^ has at least two explanations; firstly, we excluded individuals with prior cardiovascular disease or diabetes at baseline, in which the persistence to ACEi is augmented because ACEi have been recommended in guidelines for patients with these conditions; secondly, ACEi have been prioritized over ARBs before the release of the patent for ARBs for cost reasons. Accordingly, newer studies with similar methodology to ours show similar results.^8^^,^^24^

Beta-blockers were not included in the current study because they were recommended as a second line treatment at the time in Sweden. Initiators of that class were therefore assumed to differ in clinical characteristics from those initiating other classes to an extent that would preclude bias-minimization.

Limitations of our study include the lack of blood pressures, laboratory data and diagnosis codes from primary care. This primarily affects our ability to adhere to the inclusion criteria. To mitigate this risk, retrieved prescriptions of several different medications indicating a particular disease were used as proxies in conjunction with diagnosis codes and procedure codes gathered from the secondary and tertiary care data. For the antihypertensive drugs, we also considered the indication stated in the first prescription. This meant excluding those lacking an indication on the first prescription (about 20%) but we believe this to affect the drug classes non-differentially. Earlier Swedish data indicate no meaningful differences in baseline blood pressure between groups initiating different monotherapy options.^20^ Initiating treatment with an SPC however was associated with higher baseline blood pressure^20^ why an unbiased comparison between SPC and monotherapy options cannot be made in this study. Lastly, because only 2.7% of the population initiated treatment with an SPC (reflecting that this practice was not introduced until the 2018 ESC/ESH guidelines) comparisons with and within the SPC group should be interpreted with caution.

Strengths of this study include the study design, utilizing the trial emulation approach, only including patients that could have been subjected to any of the studied drug classes and adjusting for appropriate confounders; the 5-year pre-baseline window to ensure treatment-naïvety at initiation; the large representative study sample; the setting of universal healthcare with negligible copayment; the possibility of combining information from several national mandatory registers with minimal loss to follow-up; and sensitivity analyses supporting the result. To further minimize bias, we (unlike earlier studies) censored participants when a cardiovascular event occurred, which otherwise could have been a reason for apparently better persistence with an ACEi or ARB, since guidelines often recommend an ACEi or an ARB after such an event. Also to make sure a higher event rate in a particular drug class did not explain the differences in persistence (as a result of censoring), we considered such events an absorbing state (black in Figs. 3 and 4) providing evidence this was not the case. Lastly, the robustness of the results is supported by the supplemental non-parametric analysis, indicating that potential unmeasured or residual confounding is unlikely to change the results.

This study offers two important conclusions. First, while our results can support earlier claims that only half of the population adhere to their treatment, this is only true when just considering the proportion of the population consecutively retrieving refills of the initial drug class without any interruptions. This does not appear to reflect common medication-taking behavior. In fact, over the years, the majority of users have one or more temporary discontinuation, and when the scope is widened, at least 80% of the initiators take at least one antihypertensive with at least 80% adherence at any timepoint during the first five years after initiation. We therefore propose the new rule of 80 to replace the prior rule of halves. This gives hope to the physician treating hypertension, and implies that the focus during the first half year should be on making sure the patient is taking their medication, and thereafter on whether the patient is taking enough medication to reach their treatment goals. Second, initiating treatment with an ARB, rather than the other recommended drug classes, is followed by much better persistence to the initial drug class. As the effects on clinical outcomes of antihypertensive drugs are mediated by their blood pressure-lowering effects, with little evidence of other class effects,^28^, ^29^, ^30^ differential effects on other measures such as persistence and healthcare utilization become important.

## Contributors

Karl Laurell: Original draft, conceptualisation, visualisation, project administration, methodology, review and editing, decision to submit.

Dave Zachariah: Methodology of supplementary method, review and editing.

Sofia Ek: Formal analysis and original draft of supplementary method, review and editing.

Karin Rådholm: Review and editing, conceptualisation.

Mats Martinell: Review and editing, conceptualisation.

Erik Lampa: Methodology, formal analysis, data curation, software, accessed and verified the underlying data.

Stefan Gustafsson: Methodology, formal analysis, data curation, software, validation, investigation, accessed and verified the underlying data.

Johan Sundström: Conceptualisation, funding acquisition, methodology, resources, supervision, project administration, review and editing, decision to submit.

## Data sharing statement

R-code for analysis will be shared upon reasonable demand. Underlying register data is of sensitive nature and cannot be shared from our group but from the National Board of Health and Welfare and from Statistics Sweden.

## Declaration of interests

KL reports receiving salary as a part time researcher from region Uppsala Primary Health and Care, and a grant from the Geriatric Foundation. KR reports a lecture a year for Astra Zeneca, payment from the Swedish Research Council for expert testimony, and unpaid board membership in the Swedish Society of Hypertension, Stroke and Vascular Medicine as well as the Swedish Diabetes Society. SG reports employment at Sence Research at time of writing the manuscript. JS reports direct or indirect stock ownership in companies (Anagram kommunikation AB, Sence Research AB, Symptoms Europe AB, MinForskning AB) providing services to companies and authorities in the health sector including Amgen, AstraZeneca, Bayer, Boehringer, Eli Lilly, Gilead, GSK, Göteborg University, Itrim, Ipsen, Janssen, Karolinska Institutet, LIF, Linköping University, Novo Nordisk, Parexel, Pfizer, Region Stockholm, Region Uppsala, Sanofi, STRAMA, Takeda, TLV, Uppsala University, Vifor Pharma, WeMind. All other authors declares no competing interest.
