# Supplementary material for: Persistence to antihypertensive drug classes in uncomplicated hypertension: a nationwide Swedish cohort study
Source: eClinicalMedicine. 2025 Dec 19;91:103696. doi: 10.1016/j.eclinm.2025.103696 (PMC12853357; doi:10.1016/j.eclinm.2025.103696)

Supplement

[Trial emulation 2](#_Toc208221710)

[Table S1. Inclusion criteria 2](#_Toc208221711)

[Table S2. Exclusion criteria 3](#_Toc208221712)

[Figure S1. Directed acyclic graph 6](#_Toc208221713)

[Observed (unadjusted) results 7](#_Toc208221714)

[Figure S2. Observed Class persistence 7](#_Toc208221715)

[Figure S3. Observed Therapy persistence 8](#_Toc208221716)

[Additional analyses 9](#_Toc208221717)

[Figure S4. Changes in treatment during the five-year follow-up 9](#_Toc208221718)

[Figure S5. Time to retrieval of second dispensation 10](#_Toc208221719)

[Figure S6. Time to retrieval of third dispensation 11](#_Toc208221720)

[Supplementary non parametric method 12](#_Toc208221721)

[Method 12](#_Toc208221722)

[Results 12](#_Toc208221723)

[Figure S7. Reliability diagram 12](#_Toc208221724)

[Figure S8. PDAT during the first year 13](#_Toc208221725)

[References 13](#_Toc208221726)

[Results modelled with different values on confounders 14](#_Toc208221727)

[Figure S9. Class persistence modelled by age 14](#_Toc208221728)

[Figure S10. Class persistence modelled by income 15](#_Toc208221729)

[Figure S11. Class persistence modelled by time for initiation 16](#_Toc208221730)

[Figure S12. Class persistence modelled by birth country/region 17](#_Toc208221731)

[Figure S13. Class persistence by highest education 18](#_Toc208221732)

[Figure S14. Class persistence modelled by marital status 19](#_Toc208221733)

[Figure S15. Class persistence modelled by obesity 20](#_Toc208221734)

[Figure S16. Therapy persistence modelled by age 21](#_Toc208221735)

[Figure S17. Therapy persistence modelled by income 22](#_Toc208221736)

[Figure S18. Therapy persistence modelled by time for initiation 23](#_Toc208221737)

[Figure S19. Therapy persistence modelled by birth country 24](#_Toc208221738)

[Figure S20. Therapy persistence by highest education 24](#_Toc208221739)

[Figure S21. Therapy persistence modelled by marital status 26](#_Toc208221740)

[Figure S22. Class persistence without censoring CVD 27](#_Toc208221741)

[Figure S23. Therapy persistence without censoring CVD 27](#_Toc208221742)

[Sensitivity analyses 28](#_Toc208221743)

[Figure S24. Class persistence 80d 28](#_Toc208221744)

[Figure S25. Therapy persistence 80d 28](#_Toc208221745)

[Figure S26. Class persistence 100d 29](#_Toc208221746)

[Figure S27. Therapy persistence 100d 29](#_Toc208221747)

[Class persistence when changing to SPC containing original drug class ≠ class persistent 30](#_Toc208221748)

[Figure S28. Class persistence. SPC ≠ class persistent 30](#_Toc208221749)

## Trial emulation

### Table S1. Inclusion criteria

| Target trial | Emulated trial |
| --- | --- |
| *Inclusion criteria* | *Inclusion criteria modified for observational data* |
| 1. Male or female aged ≥40 years | 1. Male or female aged ≥40 years on the date of inclusion. |
| 2. Planned treatment with one of the following blood pressure lowering drugs for hypertension in a single pill:  ARB  ACEi  CCB  TD/TD like  ACEi + TD The inclusion and exclusion criteria and its relation to the a target trial (using the trial emulation framework) is described in detail in table S1 and S2.  ACEi + CCB  ARB + TD  ARB + CCB | 2.  a. A retrieved prescription of ARB, ACEi, CCA, TD in monotherapy or a single combination pill containing ACEi + diCCA, ACEi+ TD, ARB+ TD or ARB + CCB between 1 of January 2011 and 31 of December 2018 in the national prescription register, *ATC:*  *C09C (ARB)*  *CO9A (ACEi)*  *C08CA (CCB)*  *C03AA (TD), C03BA04, C03BA11 (thiazide-like/chlortalidone + Indapamide)*  *C09BA (ACEi + TD)*  *C09BB (ACEi + CCB)*  *C09DA (ARB + TD)*  *C09DB (ARB + CCB)*  *b. Dosage-information of index prescription stating treatment for hypertension.*  *c. Prescription information not stating PRN/as needed* |
| 3. Treatment naïve. | 3. No prior retrieved prescription in the 5 years prior to inclusion of the following ATC in the national prescription register:  C09 (RAS)  C07 (BB)  C08 (CCB)  C03 (diuretics)  C02 (Other blood pressure-lowering drugs) |
| 4. No concurrent treatment of any other blood-pressure lowering drug. | 4. More than one pill of antihypertensive retrieved at the date of inclusion using the following ATC:  C09 (RAS)  C07 (BB)  C08 (CCB)  C03 (diuretics)  C02 (Other blood pressure-lowering drugs) |
| 5. No prior inclusion in current study | 5. No prior inclusion in current study |

### Table S2. Exclusion criteria

| Target trial | Emulated trial |
| --- | --- |
| *Exclusion criteria* | *Exclusion criteria modified for observational data* |
| 1.Previous or current health condition with compelling indication for specific class of antihypertensive.  Heart failure  Stroke  Ischemic heart disease  Atrial fibrillation  Diabetes mellitus  Hypertensive kidney disease/kidney failure  Peripheral artery disease  Asymptomatic atherosclerosis  Aortic aneurysm  Left ventricular hypertrophy (LVH)  Peripheral oedema | 1.  a. As indicated by ICD code as a main- or bi- diagnosis in the National patient register at the date of inclusion or earlier:  I11.0, I13.0, 13.2, I50 (heart failure)  I60-64, I§66-67, I69, G45 (stroke and TIA)  I20-25 (ischemic heart disease)  I48 (atrial fibrillation or flutter)  E10-14 (diabetes)  N18.3-N18.5, N18.9, I12-13 (renal failure stage 3 to 5 + other)  I13.1 (Hypertensive heart and renal disease with renal failure)  I15.0-1 (renovascular hypertension)  I73.9 (peripheral artery disease)  I74 (arterial embolism and thrombosis  I70 (atherosclerosis)  I71.1-9 (aortic aneurism)  I51.7 (cardiomegaly (includes LVH))  I42.0 (dilated cardiomyopathy)  I42.6 Alcoholic cardiomyopathy)  b. As indicated by retrieval of at least one prescription of the following medications during the 5 years prior to, or on the date of inclusion in the national prescription register using ATC code:  A10 (antidiabetics)  C01AA (digitalis)    c. As indicated by a medical or surgical procedure, by KVÅ code in the national patient register at the date of inclusion or earlier:  DR012-13, DR023-24 (peritoneal dialysis)  DR014-17, DR020, DR060-61 (Hemodialysis)  DR018 (Hemoperfusion)  DR055 (Citrate dialysis)  DR056 (Heparin free dialysis)  d. Dosage information of the index prescription indicating the reason for the prescription was heart failure  e. Dosage information of the index prescription indicating the reason for the prescription was peripheral oedema. |
| 2. Possible contraindications for specific therapies  Gout  Hyperkalemia  Hypokalemia  Hyponatremia  Angioneurotic oedema  Current treatment with medication that interacts with one or more of the study medications | 2.  a. As indicated by ICD code as a main- or bi- diagnosis in the National patient register at the date of inclusion or earlier:  M10 (gout)  E26 (hyperaldosteronism*)*  T783 (angioneurotic edema)    b. As indicated by ICD code as a main diagnosis or contributing diagnosis in the national patient register during the 1 year prior to, and on, the date of inclusion:  E87.5 (hyperkalemia)  E87.6 (hypokalemia*)*  E871B (hyponatremia)  c. As indicated by retrieval of at least two prescriptions during the 5 years prior to, and on, the date of inclusion according to ATC:  M04A (gout medication)  d. As indicated by retrieval of at least two prescriptions, during the 1 year prior to, and on, the date of inclusion according to ATC:  A12BA (potassium supplements)  C10AC01 (cholestyramine)  C10AC02 (colestipol)  N03A (anti-epileptics)  J02AB (Imidazole derivates-antimycotic for systemic use)  J02AC (Triazole and tetrazole derivate - antimycotic for systemic use)  J05AE03 (Protease inhibitor-HIV drug)  V03AX03 (cobicistat-HIV drug)  L02BB (Antiandrogen)  J04AB02 (Rifampicin) |
| 3. A health condition that may significantly affect adherence, blood pressure-effects or side-effects.  Dementia  A history of serious mental conditions  Chronic hepatic disease  Alcohol mediated hepatic disease  Active Malignant neoplasm  Active thyroiditis or thyrotoxicosis  Acute or serious hepatic disease (not necessarily chronic) in the last year  Active endocrine disease that effects blood pressure  Possible secondary hypertension not excluded earlier. | 3.  a. As indicated by ICD code as a main- or bi- diagnosis in the National patient register at the date of inclusion or earlier.  F00-03 (dementia)  F20 -29 (Schizophrenia, schizotypal disorders and delusional disorder)  F60-61 (personality disorder)  K74 (Hepatic fibrosis, sclerosis or cirrhosis)  K73 (Chronical hepatitis not classified in another location)  K70 (hepatic disease because of alcohol)  b. As indicated by a main diagnosis of ICD code in the national patient register in the 1 year before, and on, the date for inclusion in study:  C00-43 and C45-97 (malignant neoplasm)  E05 (thyrotoxicosis), E06 (thyroiditis)  K71 (toxic hepatic disease)  K72 (acute and chronic hepatic failure)  K75 (other inflammatory liver diseases)  K76 (other liver disease)  K77 (liver disorders in diseases classified elsewhere)  E27 (Disorders of the adrenal gland, including Addison but not Cushing syndrome.)  I15.2 (hypertension secondary to endocrine disorders)  I15.8-9 (other secondary hypertension/secondary hypertension unspecified)  I77.3 (Arterial fibromuscular dysplasia) |
| 4. Medication received through pre-dispensed drug packages/APO-dos | 4. The first index prescription is retrieved i pre-dosage packages |

### Figure S1. Directed acyclic graph

Pregnancy was unobserved but the primary treatment for hypertension in pregnant woman is a b-blocker and only 4.8% of children in Sweden are born by a mother of 40 years older. In conjunction with our criteria to be included in the study (age ≥ 40 years and no b-blocker) this means unmeasured bias for this reason will be minimal.

## Observed (unadjusted) results

### Figure S2. Observed Class persistence


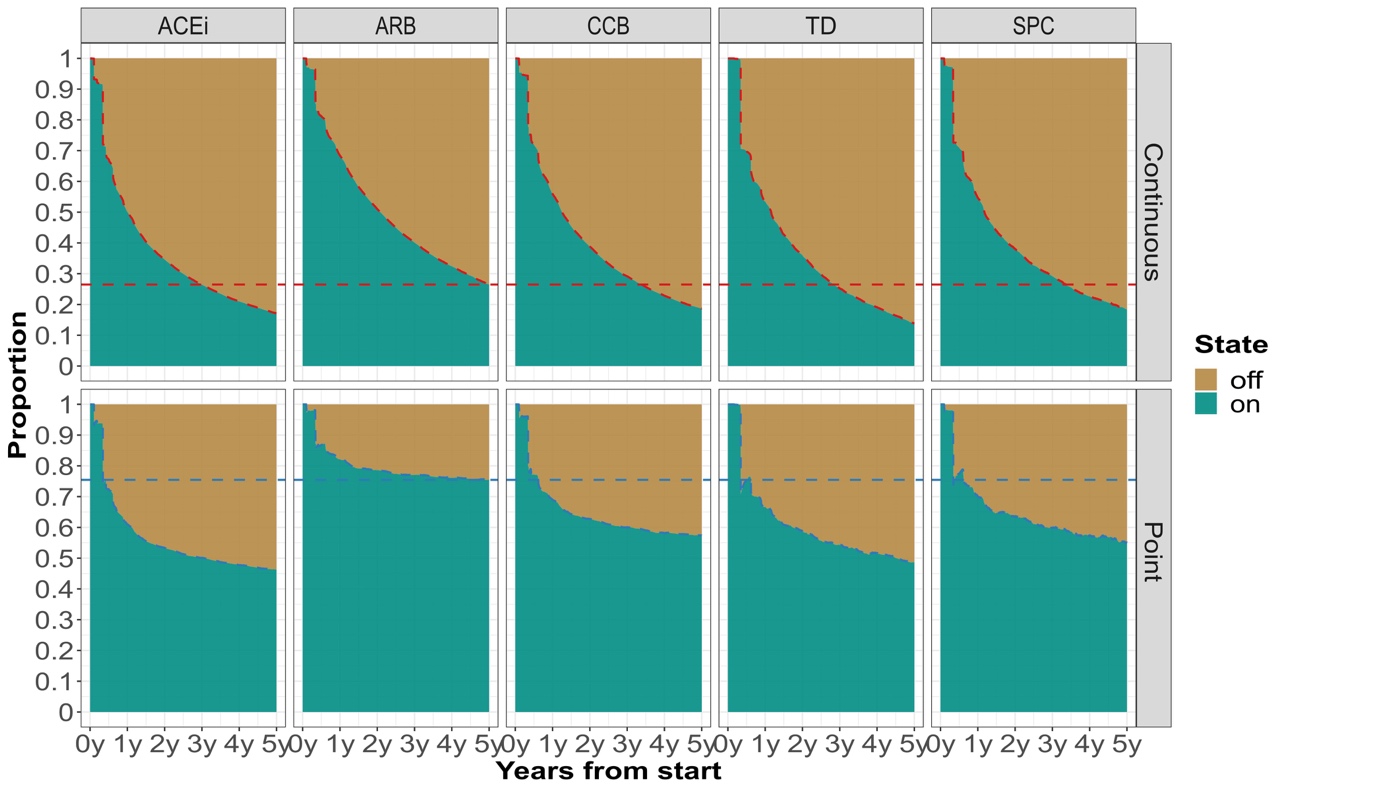


Unadjusted values for class persistence. The top bar displays the proportion continuously persistent to their original drug class (continuous class persistence). The lower bar displays the proportion currently persistent to their original drug class at different time points when intermittent discontinuations are allowed (point class persistence).

###

### Figure S3. Observed Therapy persistence


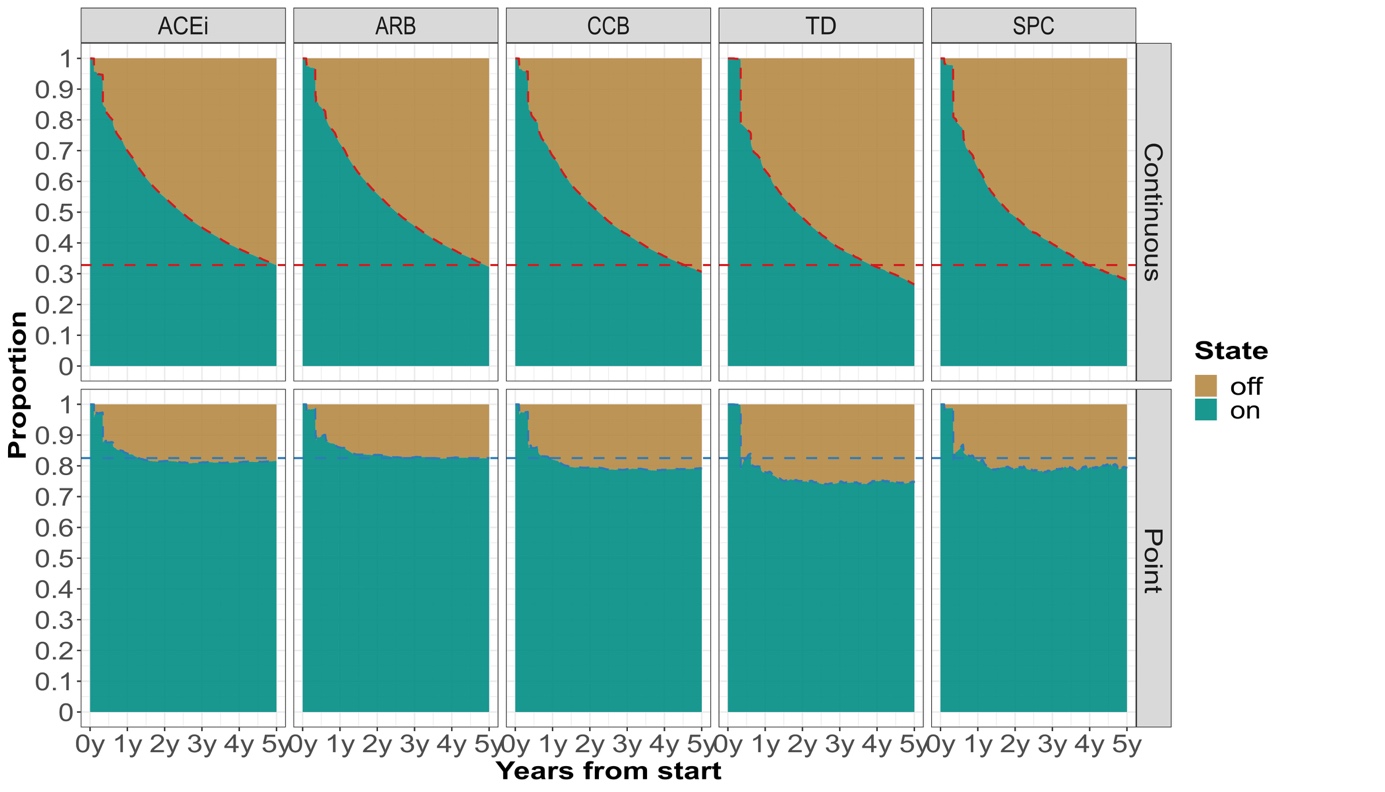


Unadjusted values for therapy persistence. The top bar displays the proportion continuously persistent to any of the of the studied antihypertensives (continuous therapy persistence). The lower bar displays the proportion currently persistent to any of the of the studied antihypertensives at different time points when intermittent discontinuations are allowed (point therapy persistence).

## Additional analyses

### Figure S4. Changes in treatment during the five-year follow-up


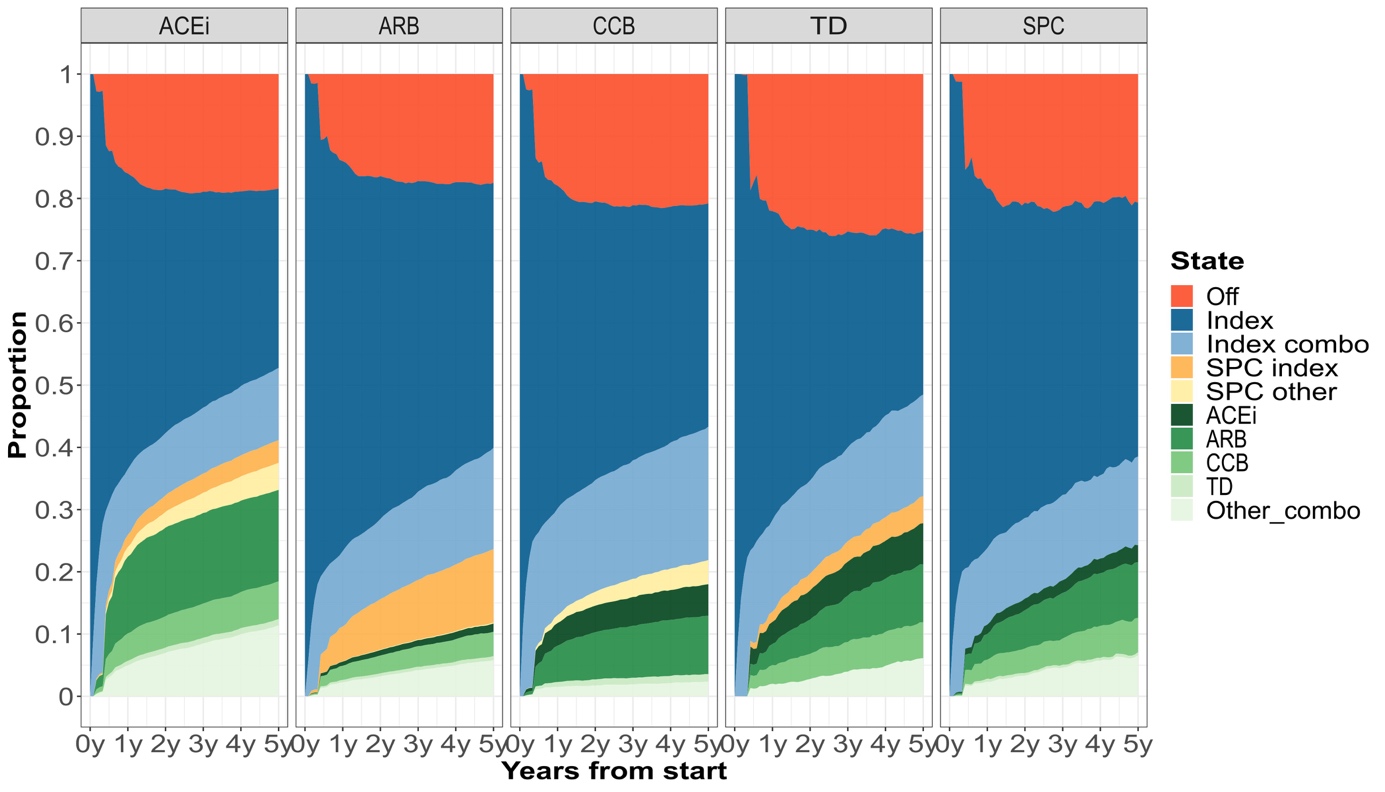


The different colors represent retrieved dispensations according to point persistence in reference to class at index. E.g a big proportion of people initiating treatment with an ACEi (column furthest to the left) later switched to an ARB, represented by the medium green field. Index = using the index drug, Index combo = using the index drug + a free combination, SPC index = using the index drug in a single pill combination, SPC other = using a single pill combination not including index drug, ACEi = using only an ACEi, ARB = using only an ARB, CCB = using only a CCB, TD = using only a TD and, other combo = using a free combination not including index drug.

### Figure S5. Time to retrieval of second dispensation

|  |
| --- |


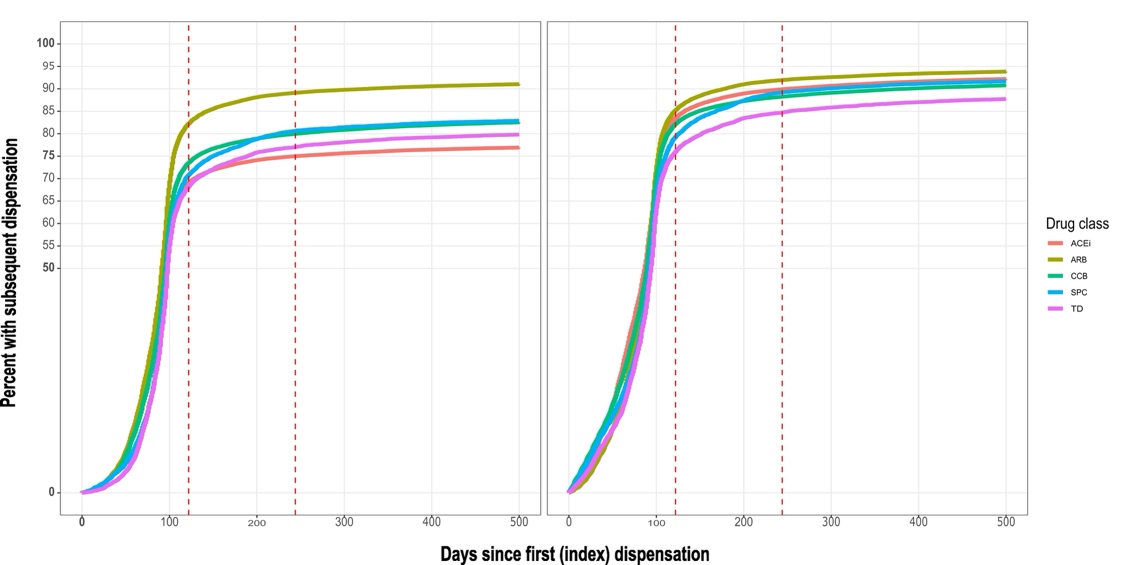


Time between the collection of the first (Time = 0) and second dispensation for the subgroup of people prescribed iterations of 98–105 pills, with a dosage of 1 pill/day. The left graph displays the proportion collecting a dispensation of the original class. The right graph displays the proportion collecting a dispensation of any of the studied antihypertensive drug classes.

### Figure S6. Time to retrieval of third dispensation


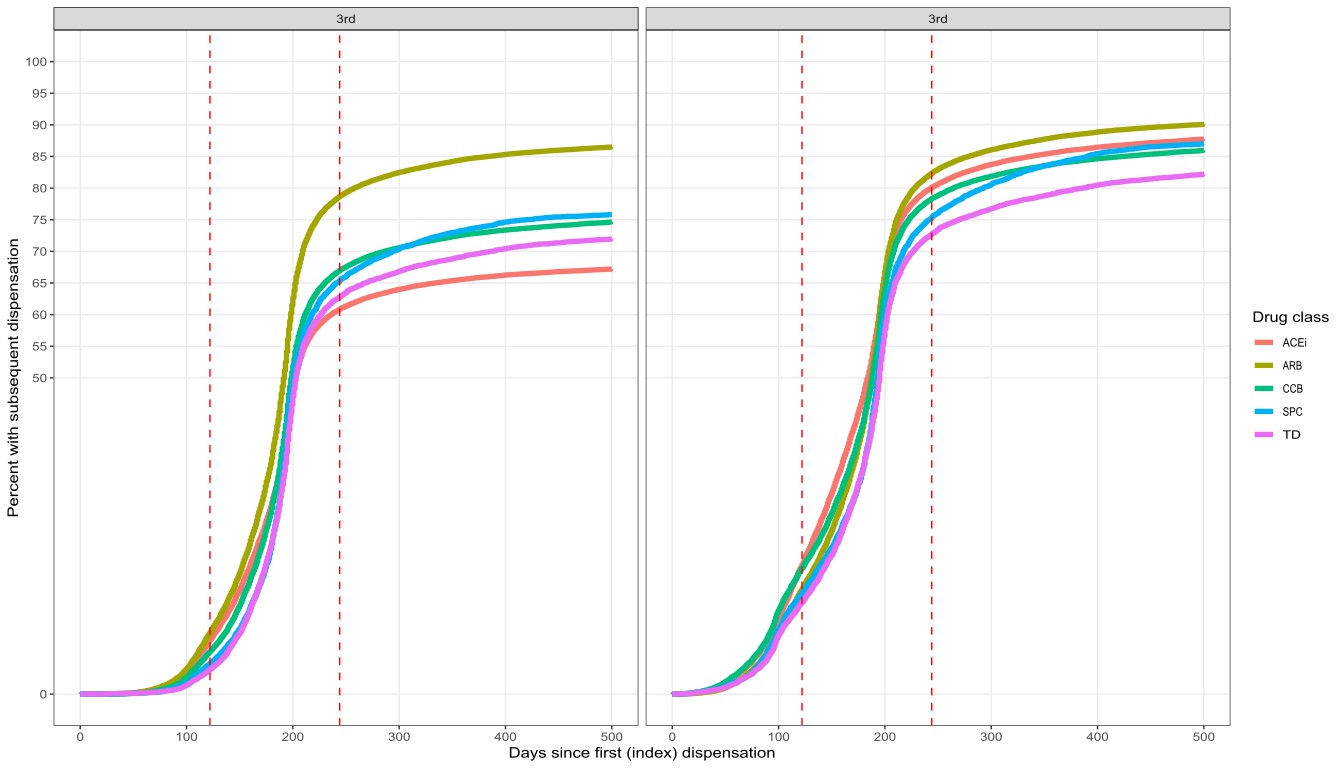


Time between the collection of the first (Time = 0) and third dispensation for the subgroup of people prescribed iterations of 98–105 pills, with a dosage of 1 pill/day. The left graph displays the proportion collecting a dispensation of the original class. The right graph displays the proportion collecting a dispensation of any of the studied antihypertensive drug classes.

## Supplementary non parametric method

### Method

To draw robust inferences, we employ an additional nonparametric method that accounts for unmeasured confounding and general misspecification of the treatment assignment models. Our focus is on inferring the proportion of days adherent to treatment (PDAT) during the first year that would result from assigning any particular drug class. We calculate adherence the same manner as described in the main analysis for the patients who continued obtaining dispensations of the index drug class (the patients that were defined as “class persistent” in the main analysis). Unlike the main analysis only patients with at least 1-year follow-up were included and patients were not censored when a cardiovascular event occurred.

This additional method is based on inverse probability weighting (IPW) where propensity scores are used to adjust for the distribution shift from past observational data to a future treatment assignment. To estimate the propensity scores from data, a tree-based ensemble model trained by XGBoost (1Chen and Guestrin, 2016) is used. Careful covariate selection and domain expertise are needed to mitigate plausible sources of confounding and similar to the main analysis, we use the following confounders: age, sex, obesity, birth country, education, income and marital status. However, to actually certify robustness of our inferences, we allow the true treatment assignment odds, based on the unknown propensity scores, to diverge from the nominal odds, obtained from the estimated propensity scores, by a factor $\Gamma\geq1$ similar to Tan, 2006 (2). That is,

$\frac{1}{\Gamma} \leq\frac{\text{odds}\left( p \right)}{\text{odds}\left( \hat{p} \right)} \leq\Gamma$.

Here $p$ is the unknown true propensity score and $\hat{p}$ is the estimated propensity score, which may diverge due to both modeling errors and unmeasured confounding. For example, setting $\Gamma= 2$ means that the causal inferences we draw remain valid even if the odds of the estimated propensity scores diverge by a factor of up to 2. The weights in the IPW estimator is then altered based on the chosen $\Gamma$ to certify robustness. To calibrate $\Gamma$, we use reliability diagrams that assess how well the average estimated nominal odds match the actual average observed odds in our data (3 Ek and Zachariah, 2024), see Figure S8. Given that all curves are close to the diagonal, we can reasonably conclude that the propensity score model is sufficiently flexible.

We can now certify that the PDAT of a future patient after assigning a particular drug, denoted $\text{PDAT}_{n+1}$, is no less than a limit $\text{PDAT}_{\alpha}$, or that

$$\mathrm{PDAT}_{n+1}\geq\mathrm{PDAT}_{\alpha}$$

with a probability of at least $1 - \alpha$ . The limit $\text{PDAT}_{\alpha}$ is calculated from data using the IPW estimator for a chosen $\Gamma$. It is a function of $\alpha$ and can be visualized as a limit curve, see Figure S9. The details of the construction and properties of the limit can be found in Ek et al., 2023 (4).

The analysis were performed in Python 3.10 using the following packages: matplotlib 3.8, numpy 1.26, pandas 2.2, seaborn 0.13, scikit-learn 1.4, scipy 1.13 and xgboost 2.0.

### Results

The $\text{PDAT}_{\alpha}$ limit curve for the first year of each assigned drug is shown in Figure S9 for both $\Gamma= 1$ and  $\Gamma= 2$. In the implausible case of neither modeling errors nor confounding ($\Gamma= 1$), we see that up to the 90%-level, ARB achieves a limit $\text{PDAT}_{\alpha}$ that is substantially higher than that of any alternative drug. Under $\Gamma= 2$, the limits that can be certified under unmeasured confounding drop notably. However, ARB still achieves substantially higher $\text{PDAT}_{\alpha}$ than any alternative, up to the 80%-level. This robustness result thereby corroborates the main results. A Python implementation for processing the data is available at <https://github.com/sofiaek/evaluation-pdat>.

### Figure S7. Reliability diagram


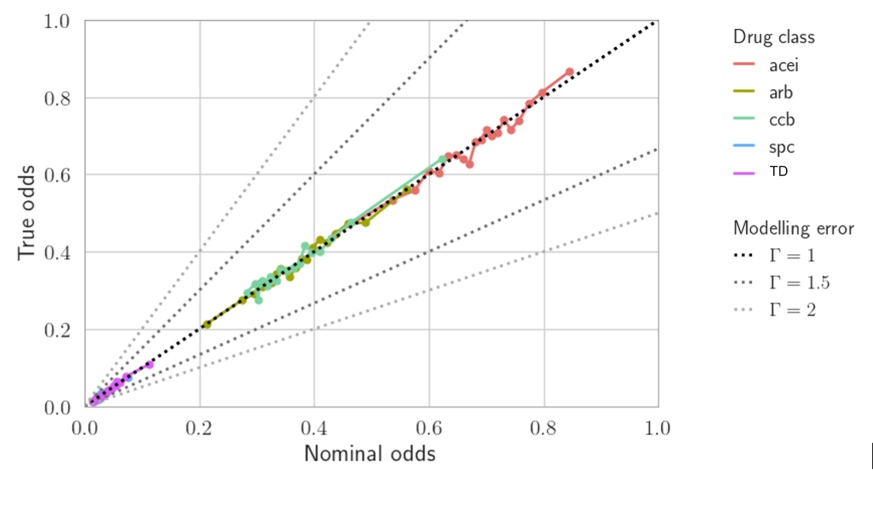


Reliability diagram comparing average estimated nominal odds from the propensity score models to average observed odds in the data, with results for each drug calculated across 20 bins.

### Figure S8. PDAT during the first year


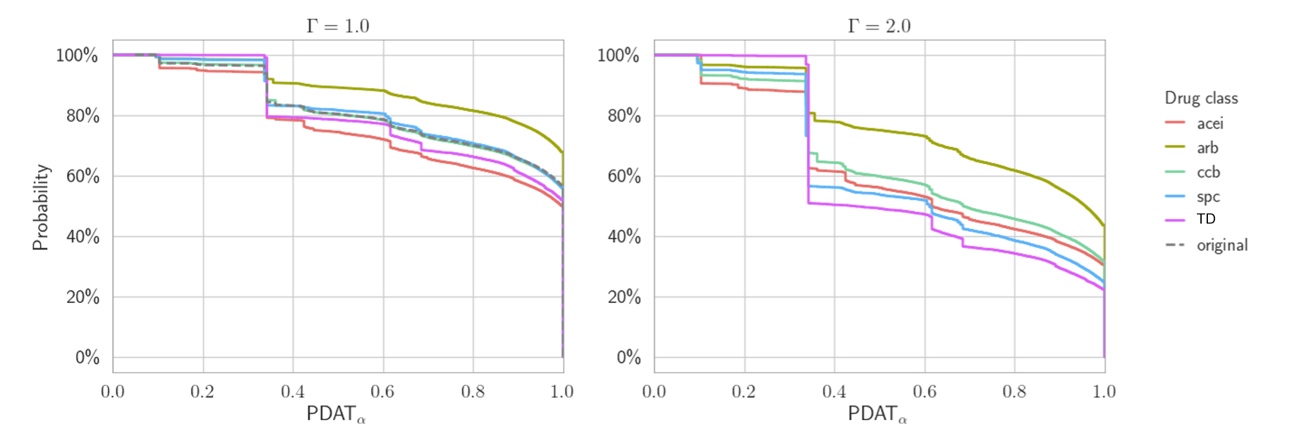


Probability of exceeding $\mathrm{PD}\mathrm{AT}_{\alpha}$ during the first year in a population under different assignments of drugs. Left: Limit curves assuming a perfectly specified treatment odds model ($\Gamma= 1$). The dotted curve indicates the actual treatment assignments used in the observational data. Right: Limit curves that are valid even when treatment odds are misspecified by a factor of 2 ($\Gamma= 2$), which includes potential confounding. The credibility of the assumptions increases with the degree of treatment odds misspecification $\Gamma$, while the informativeness of the inferences decreases accordingly.

### References

1. Chen T, Guestrin C. Xgboost: A scalable tree boosting system. Proceedings of the 22nd ACM SIGKDD International Conference on Knowledge Discovery and Data Mining. 2016:785–794.
2. Tan Z. A distributional approach for causal inference using propensity scores. Journal of the American Statistical Association. 2006;101(476):1619–37.
3. Ek S, Zachariah D. Externally valid policy evaluation from randomized trials using additional observational data. The Thirty-eighth Annual Conference on Neural Information Processing Systems. 2024.
4. Ek S, Zachariah D, Johansson FD, Stoica P. Off-policy evaluation with out-of-sample guarantees. Transactions on Machine Learning Research. 2023.

## Results modelled with different values on confounders

The model is set to median for continuous variables and to the most common level for categorical variables for the cofactor not being explored.

### Figure S9. Class persistence modelled by age

Age at initiation displayed to the right according to 2.5^th^, 25^th^, 50^th^, 75^th^ and 97.5^th^ percentile.


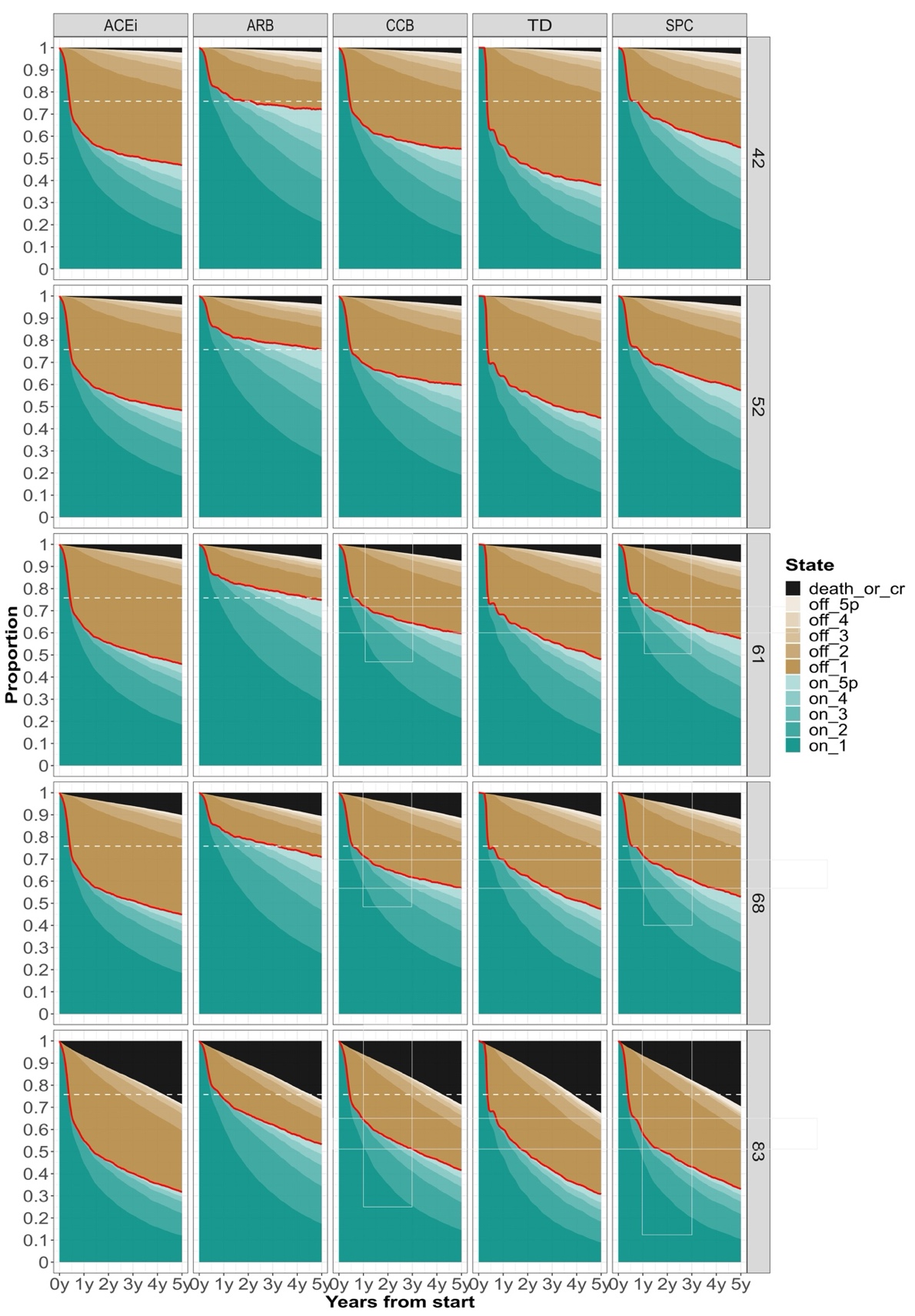


### Figure S10. Class persistence modelled by income

Year income displayed in SEK to the right according to 25^th,^ 50^th^ and 75^th^ percentile.


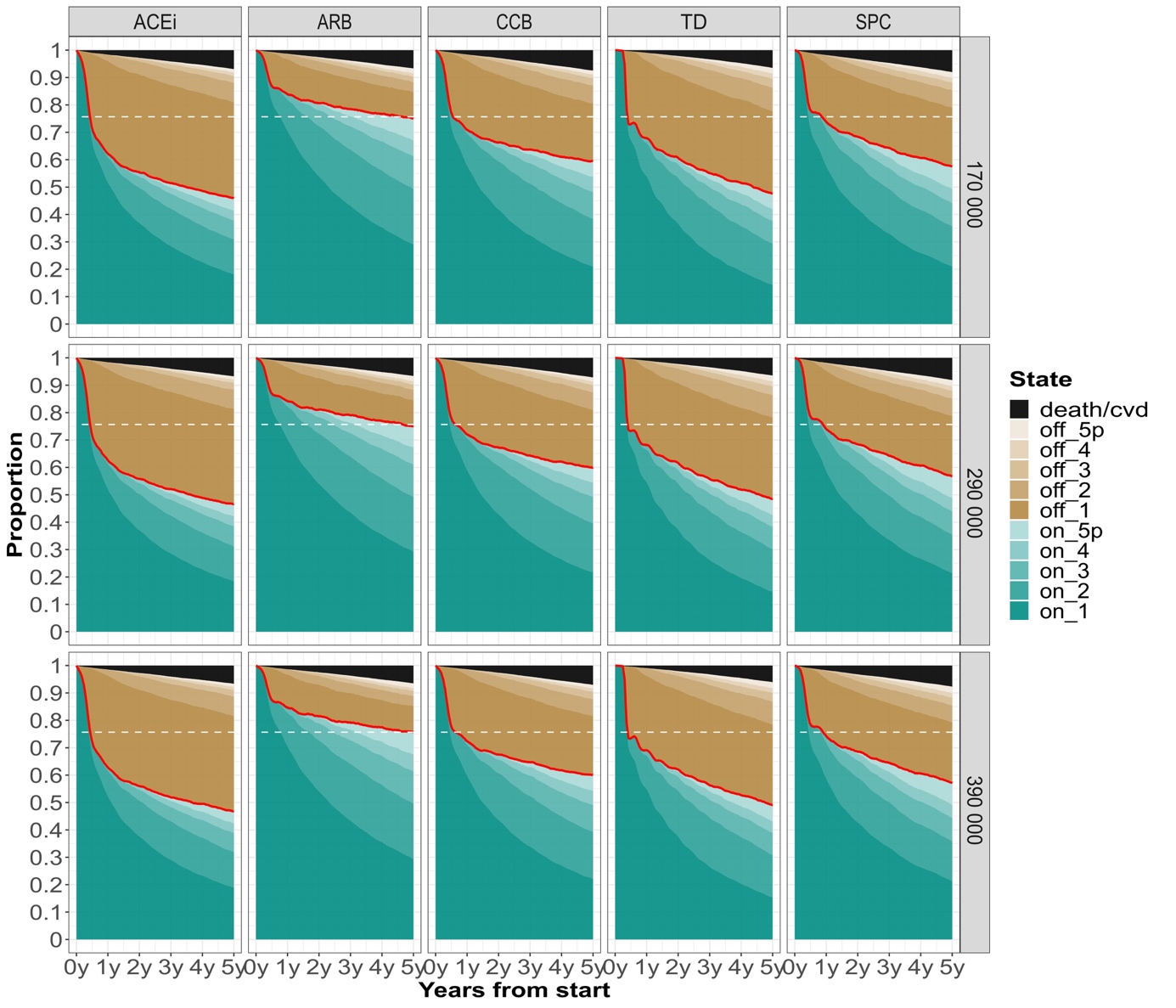


### Figure S11. Class persistence modelled by time for initiation

Time for initiation displayed to the right according to the first, median and last date for inclusion.


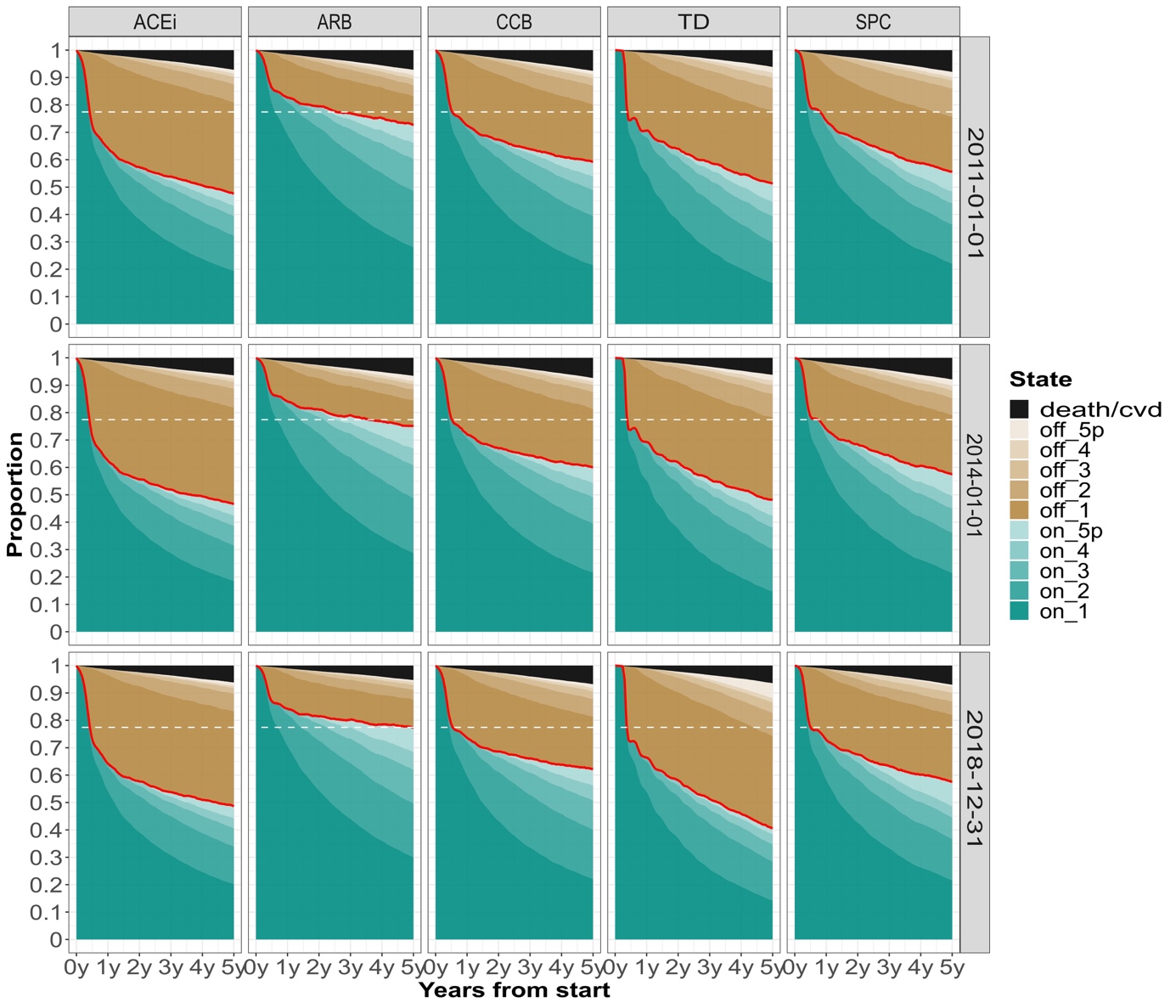


### Figure S12. Class persistence modelled by birth country/region

Birth country or region displayed to the right.


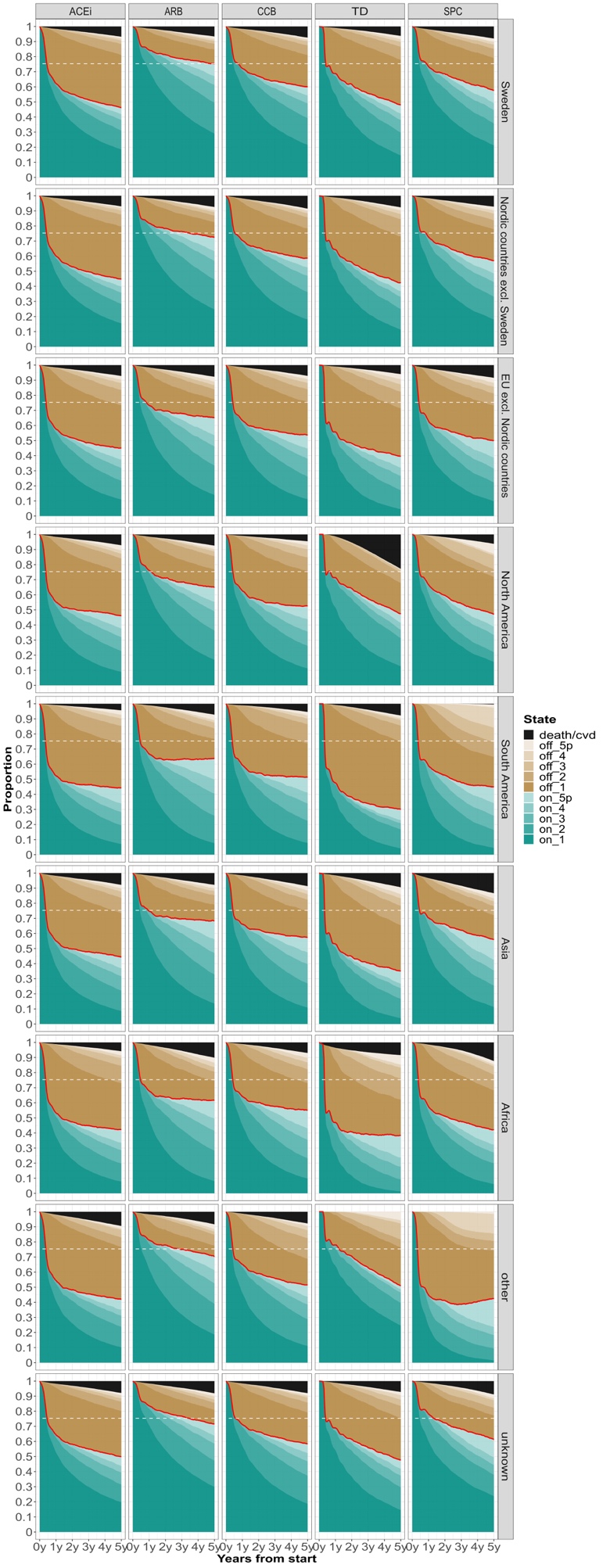


### Figure S13. Class persistence by highest education

Highest education displayed to the right.


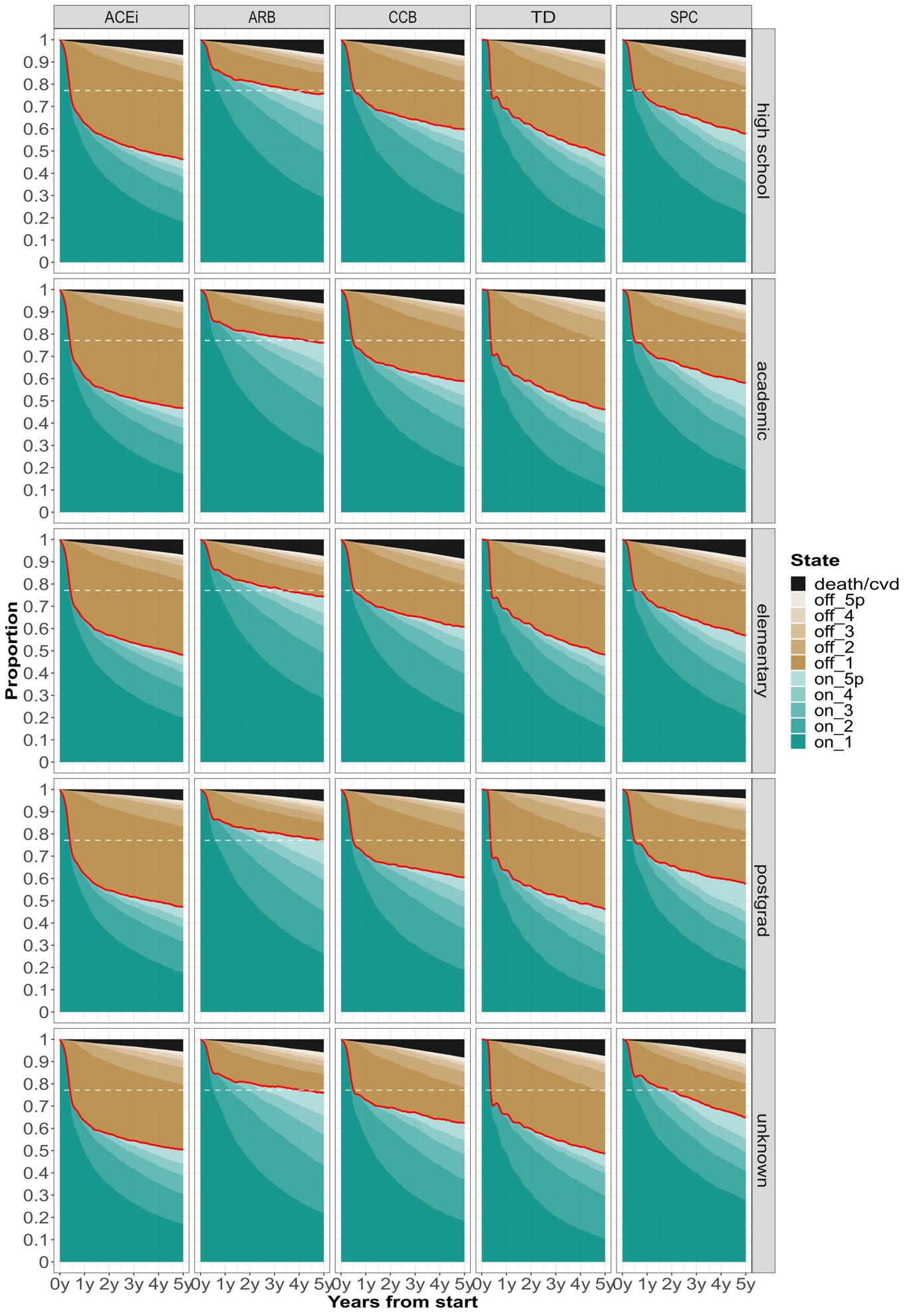


### Figure S14. Class persistence modelled by marital status

Marital status displayed to the right.


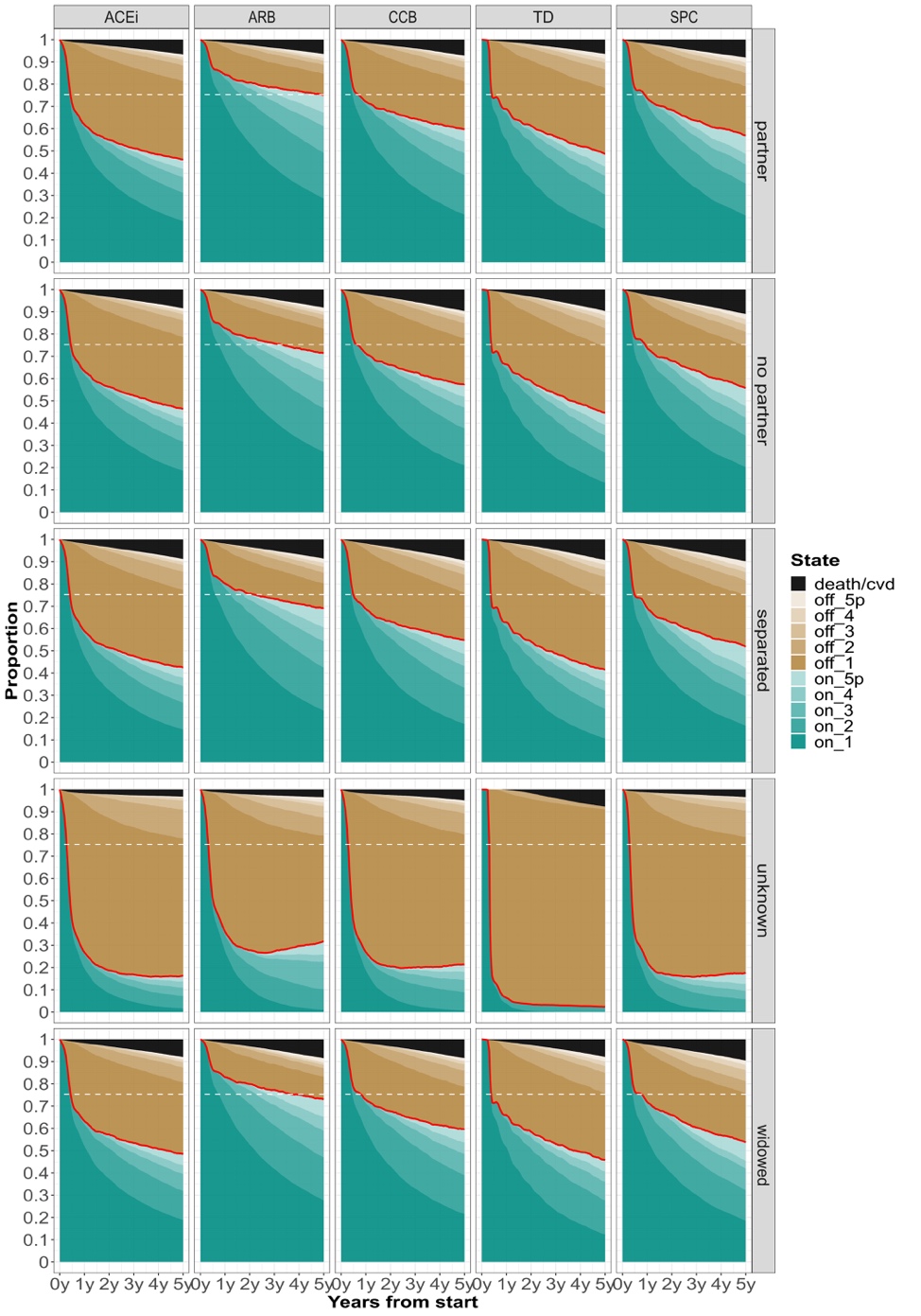


### Figure S15. Class persistence modelled by obesity

1 = obesity, 0 = no obesity

###


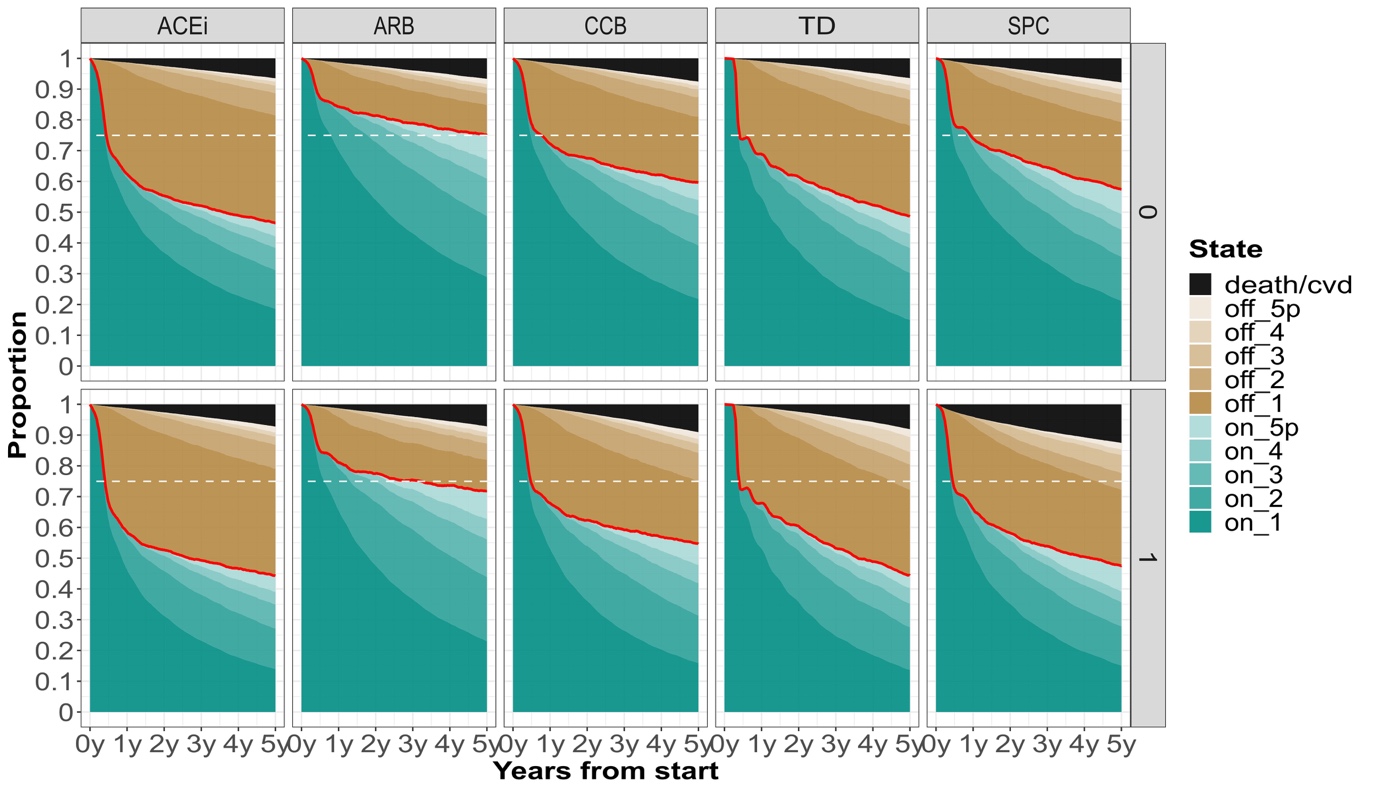


### Figure S16. Therapy persistence modelled by age

Age at initiation displayed in the right according to 2.5^th,,^ 25^th^, 50^th^, 75^th^ and 97.5^th^ percentile.


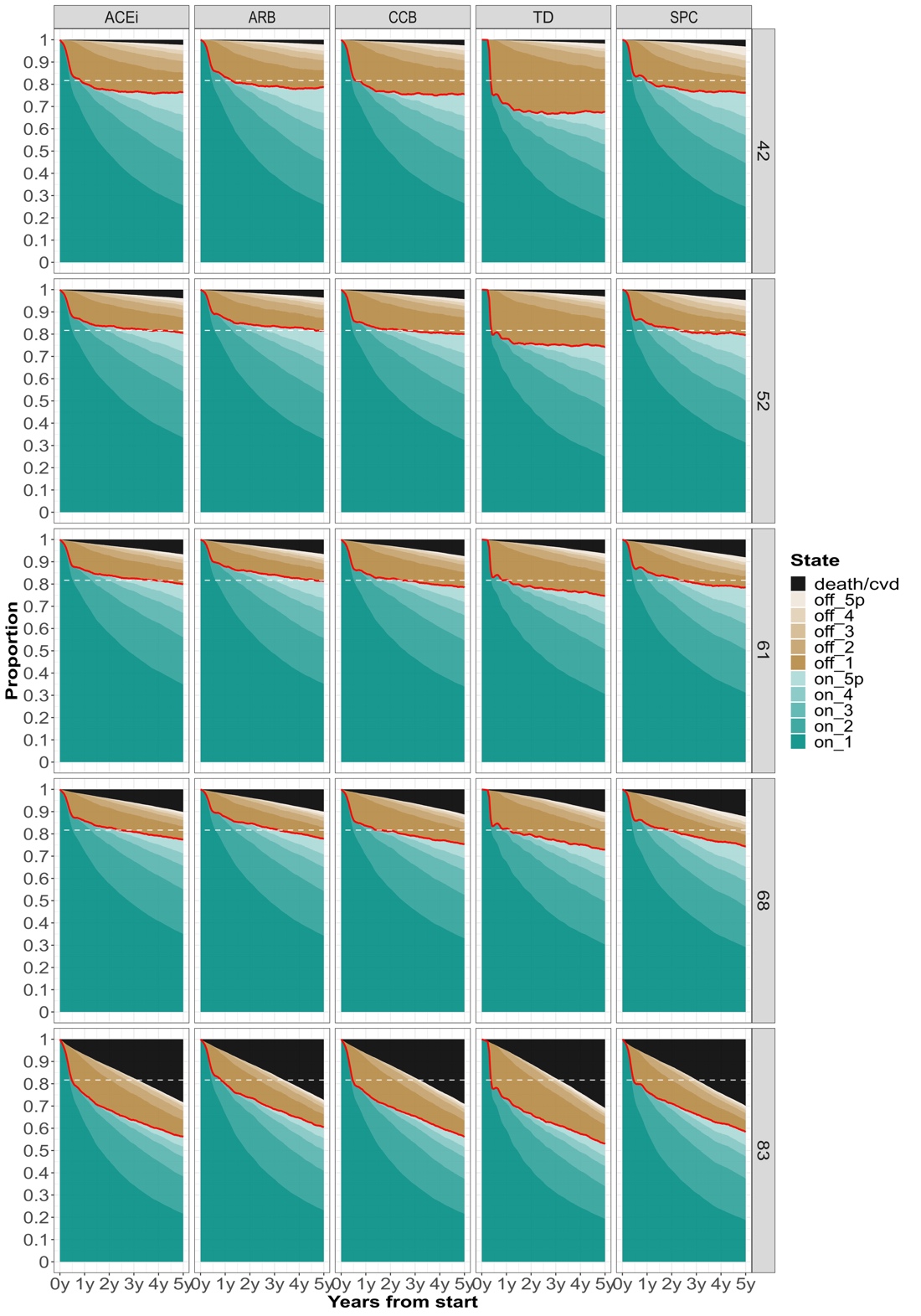


Figure S17. Therapy persistence modelled by income

Year income displayed in SEK to the right according to 25^th,^ 50^th^ and 75^th^ percentile.


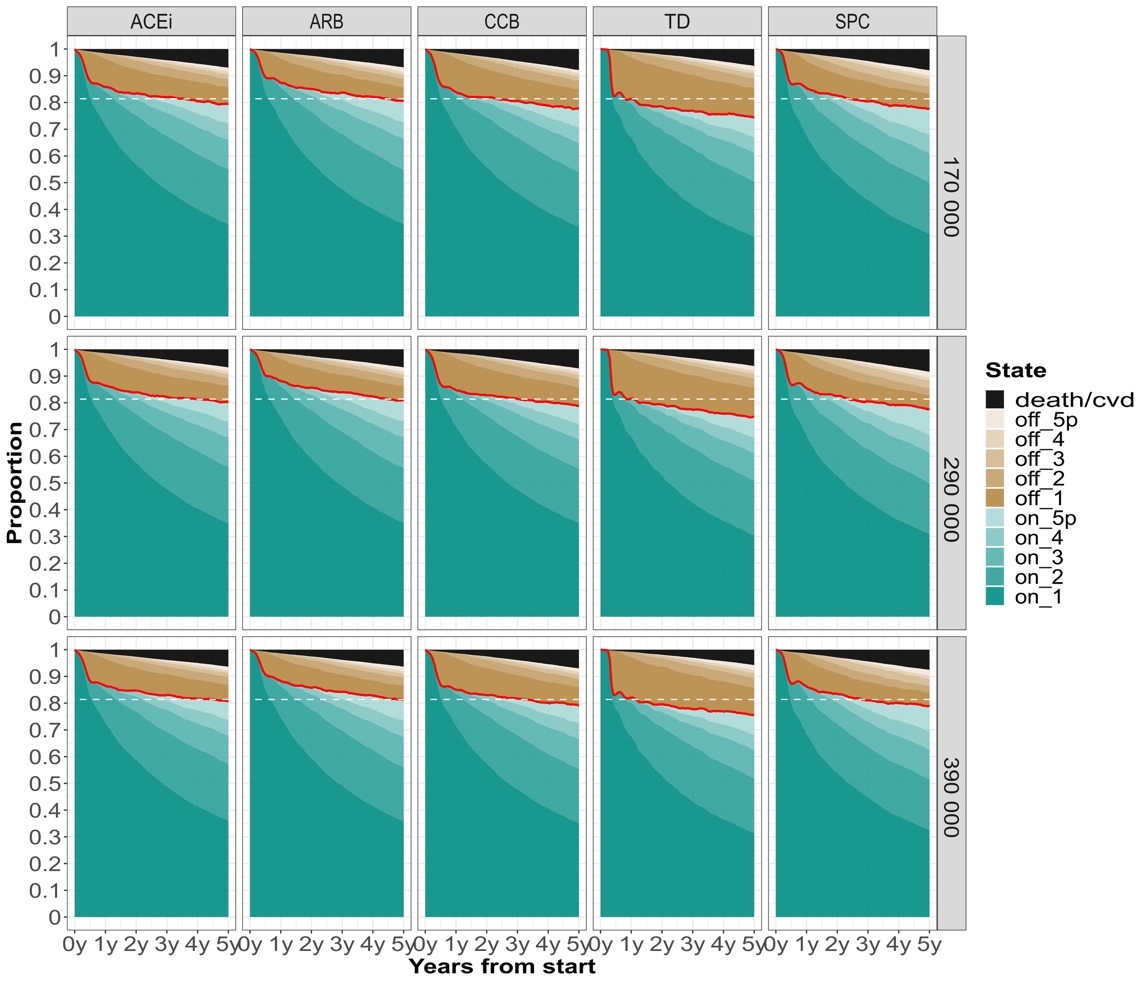


Figure S18. Therapy persistence modelled by time for initiation

Time for initiation displayed to the right according to the first, median and last date for inclusion.


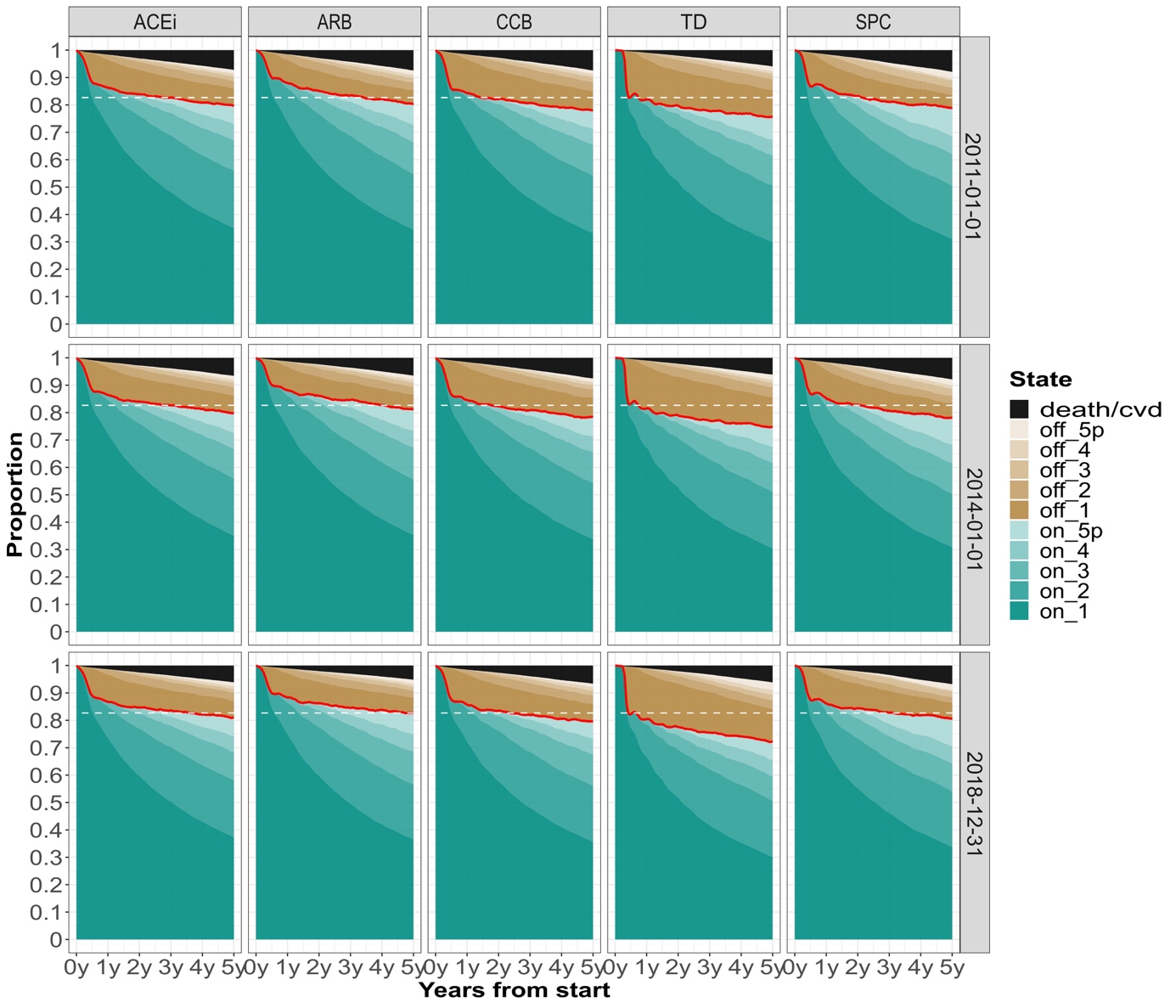


Figure S19. Therapy persistence modelled by birth country

Birth country or region displayed to the right.


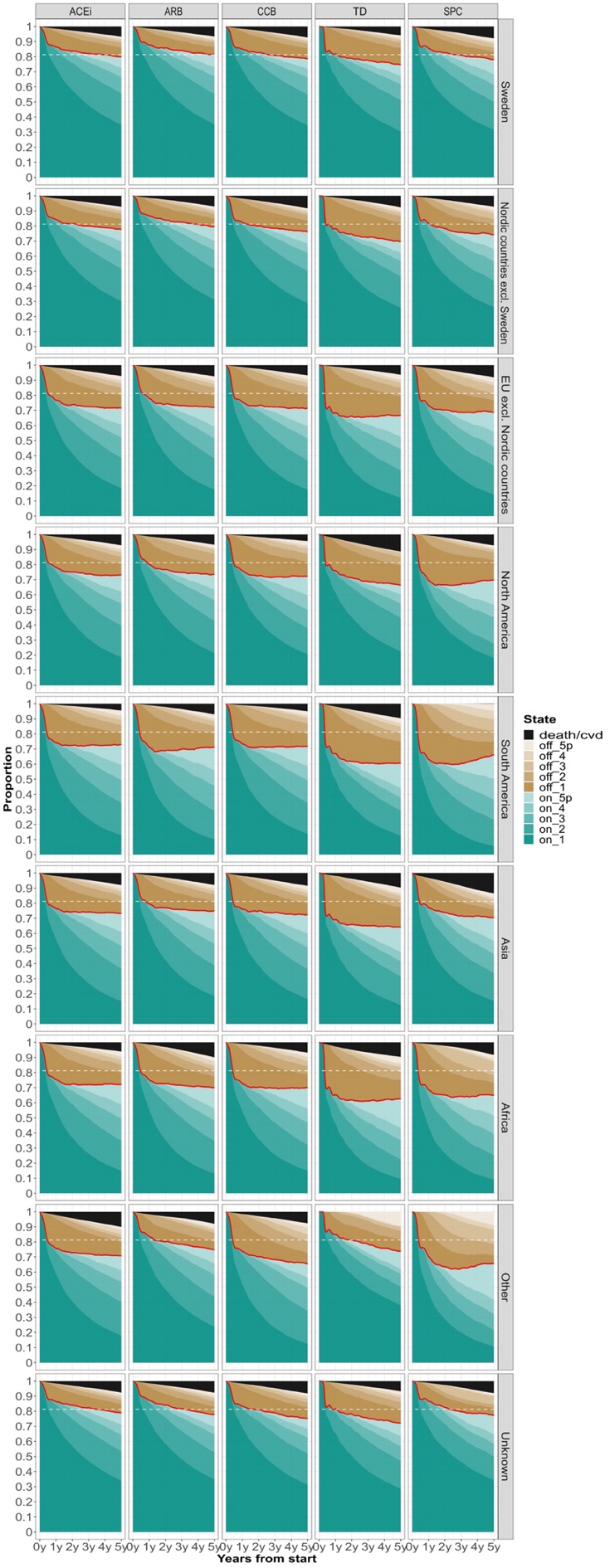


Figure S20. Therapy persistence by highest education

Highest education displayed to the right.


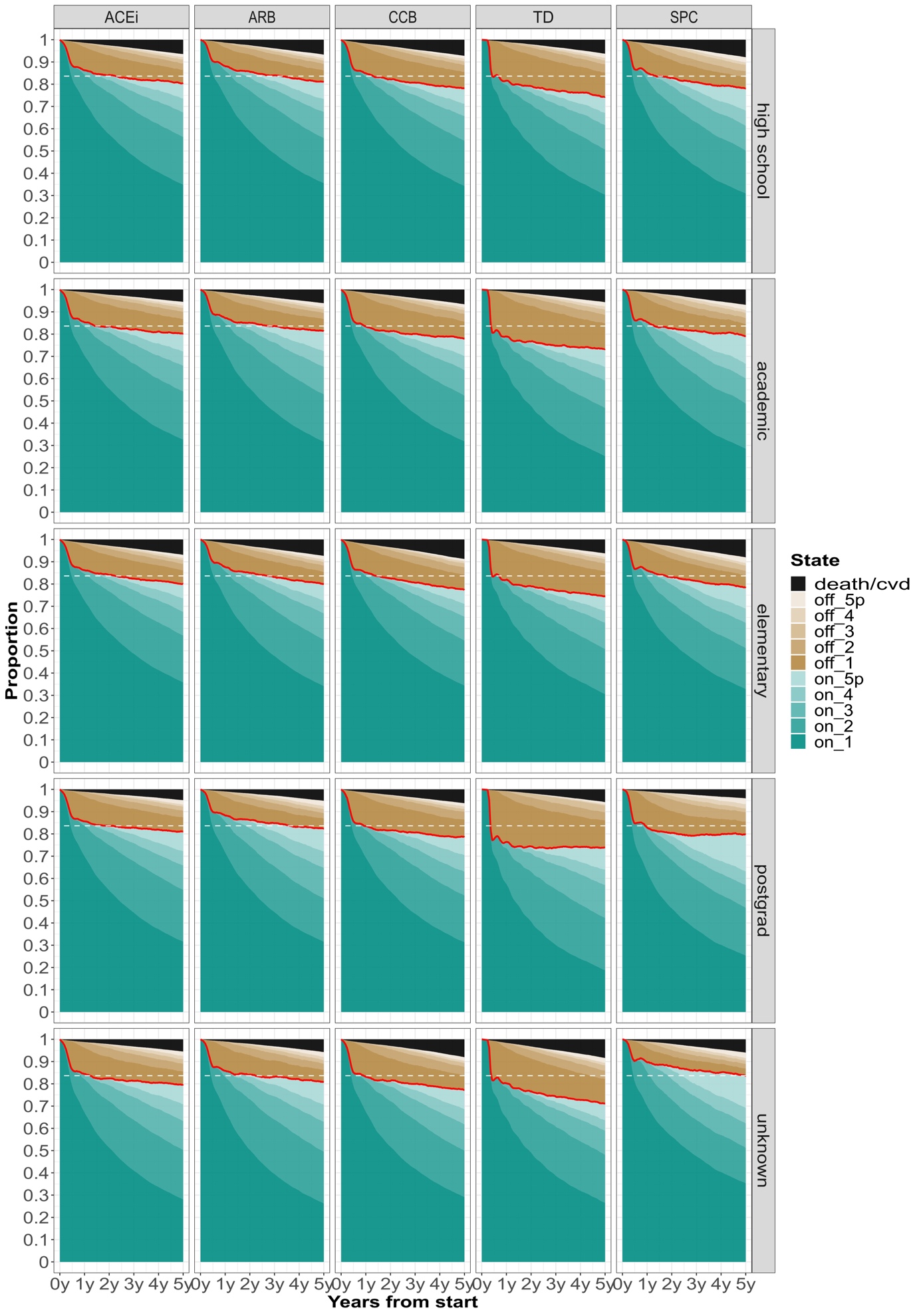


Figure S21. Therapy persistence modelled by marital status

Marital status displayed to the right.


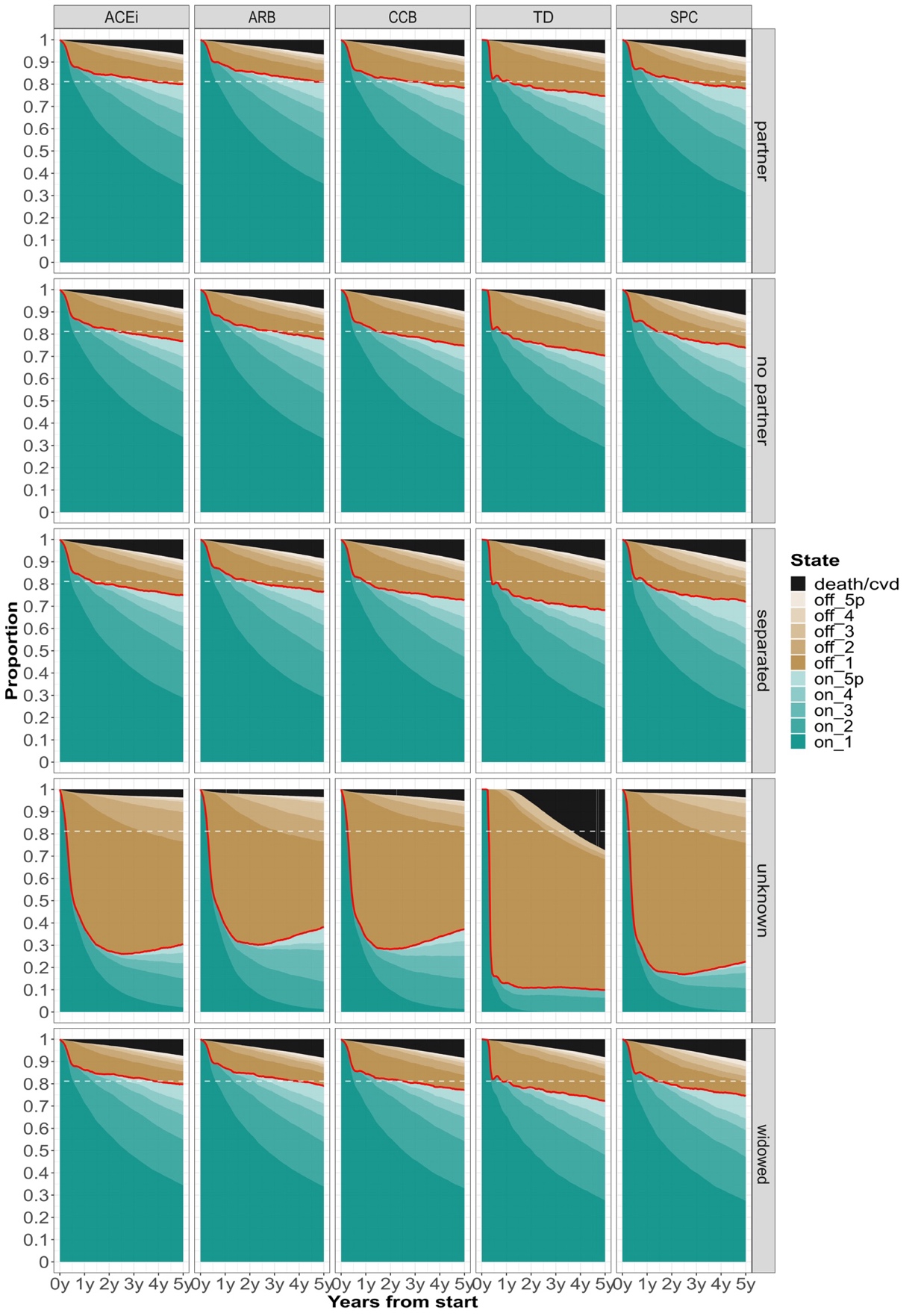
Results modelled without censoring when a non-fatal cardiovascular event occur

The model is set to median for continuous variables and to the most common level for categorical variables i.e time of initiation (2014-01-01), age (61 years), birth country (Sweden), highest education (high school), marital status (partner), total income (290 000), obesity (no) and sex (male).

### Figure S22. Class persistence without censoring CVD

Class persistence when not censoring for CVD after baseline.


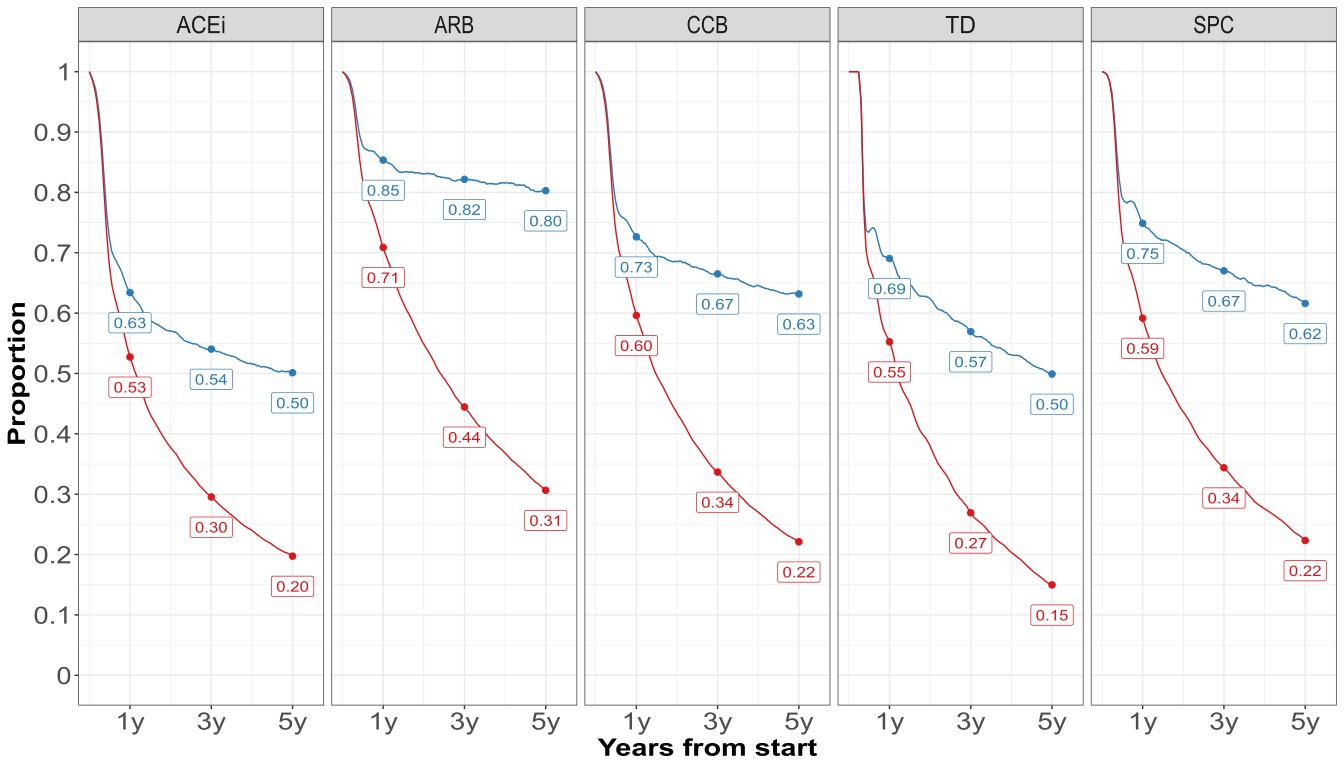


The red line illustrates continuous persistence and the blue line displays point persistence.

### Figure S23. Therapy persistence without censoring CVD

Therapy persistence when not censoring for CVD after baseline.


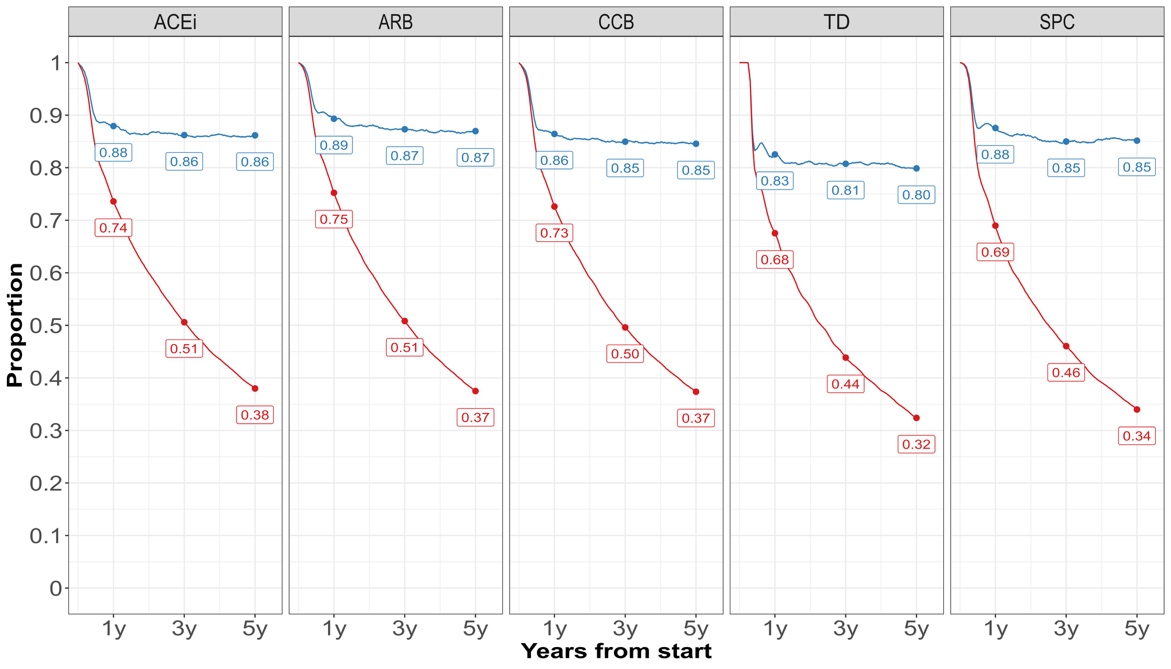


The red line illustrates continuous persistence and the blue line displays point persistence.

## Sensitivity analyses

The model is set to median for continuous variables and to the most common level for categorical variables i.e time of initiation (2014-01-01), age (61 years), birth country (Sweden), highest education (high school), marital status (partner), total income (290 000), obesity (no) and sex (male)

### Figure S24. Class persistence 80d

Class persistence assuming every dispensation covered 80 treatment days.


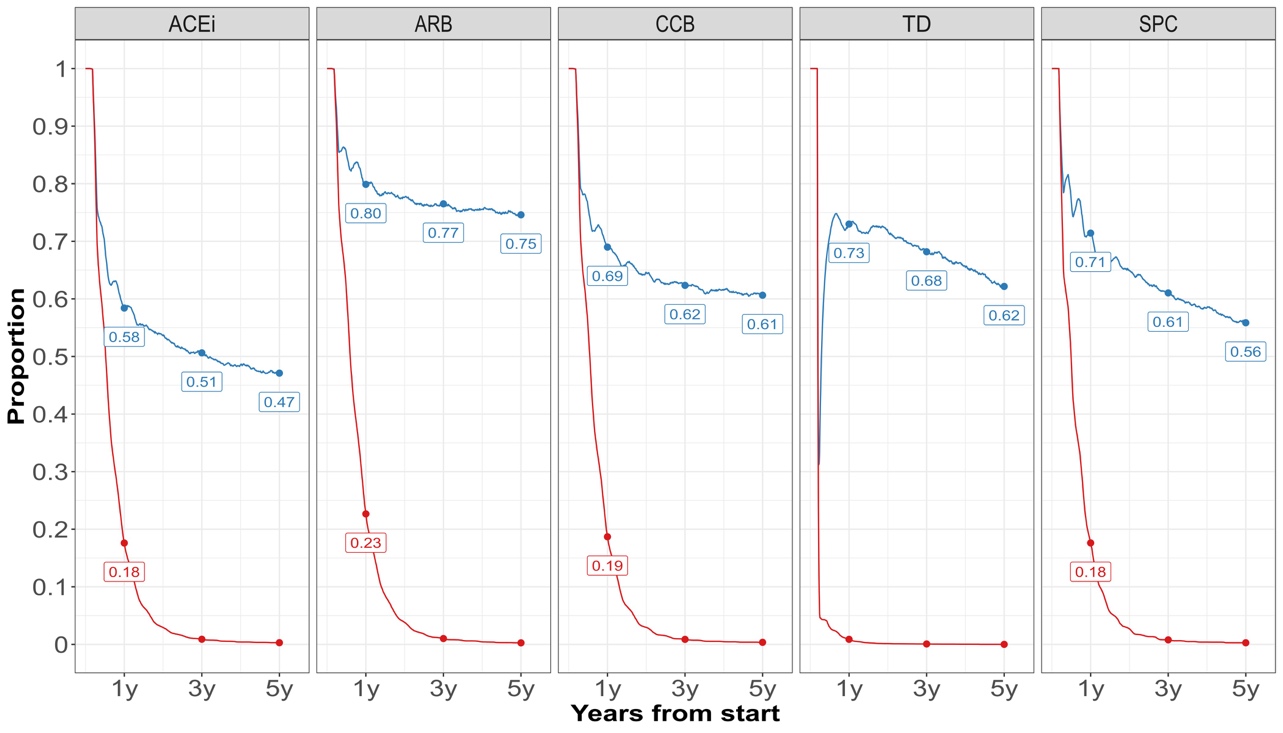


The red line illustrates continuous persistence and the blue line displays point persistence.

### Figure S25. Therapy persistence 80d

Therapy persistence assuming every dispensation covered 80 treatment days.

The red line illustrates continuous persistence and the blue line displays point persistence.


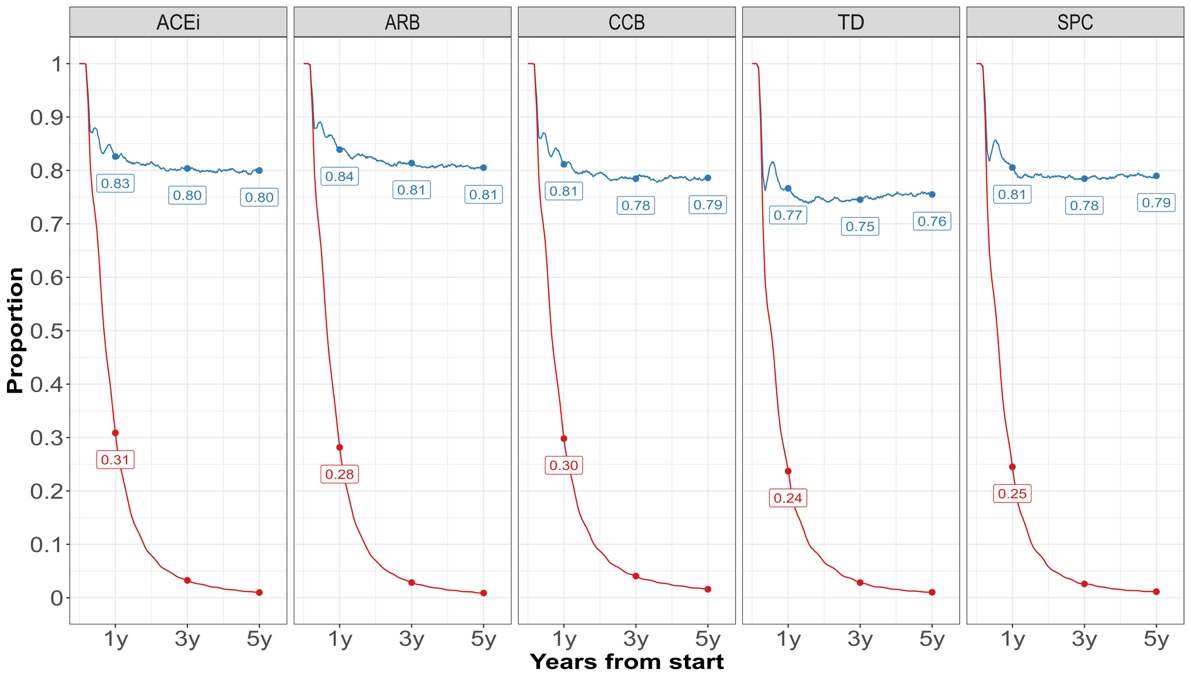


Figure S26. Class persistence 100d

Class persistence assuming every dispensation covered 100 treatment days.


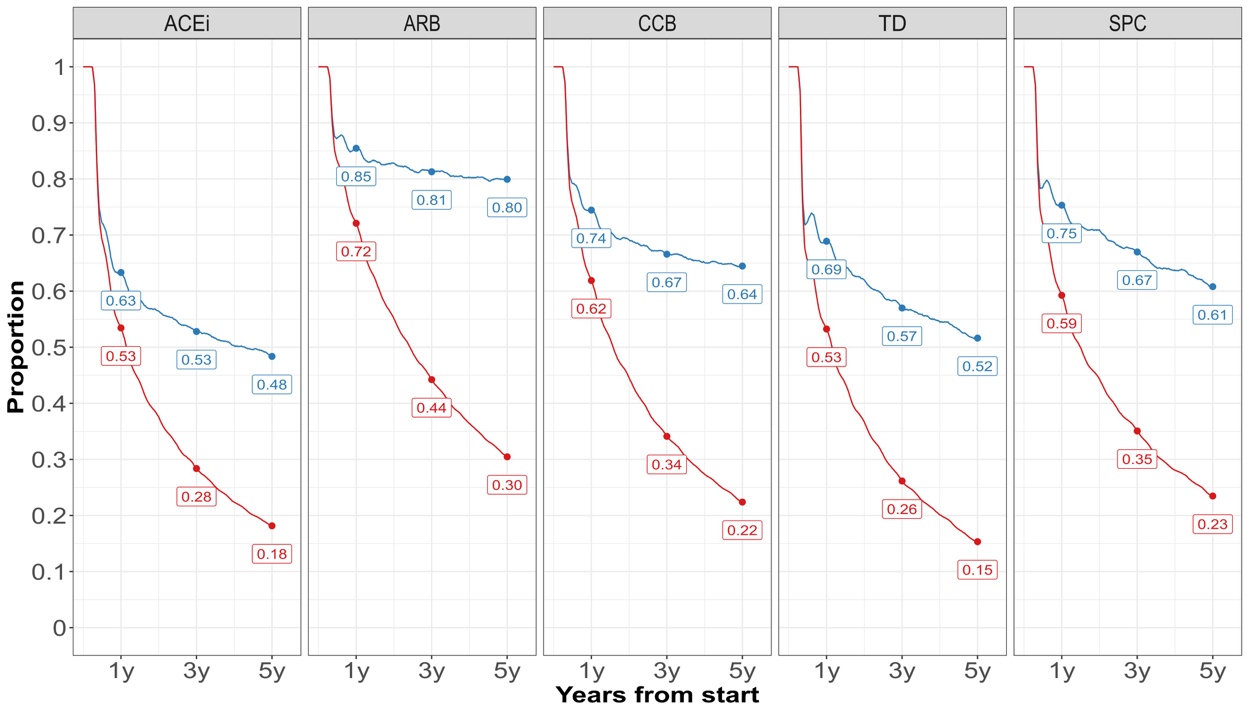


The red line illustrates continuous persistence and the blue line displays point persistence.

### Figure S27. Therapy persistence 100d

Therapy persistence assuming every dispensation covered 100 treatment days.


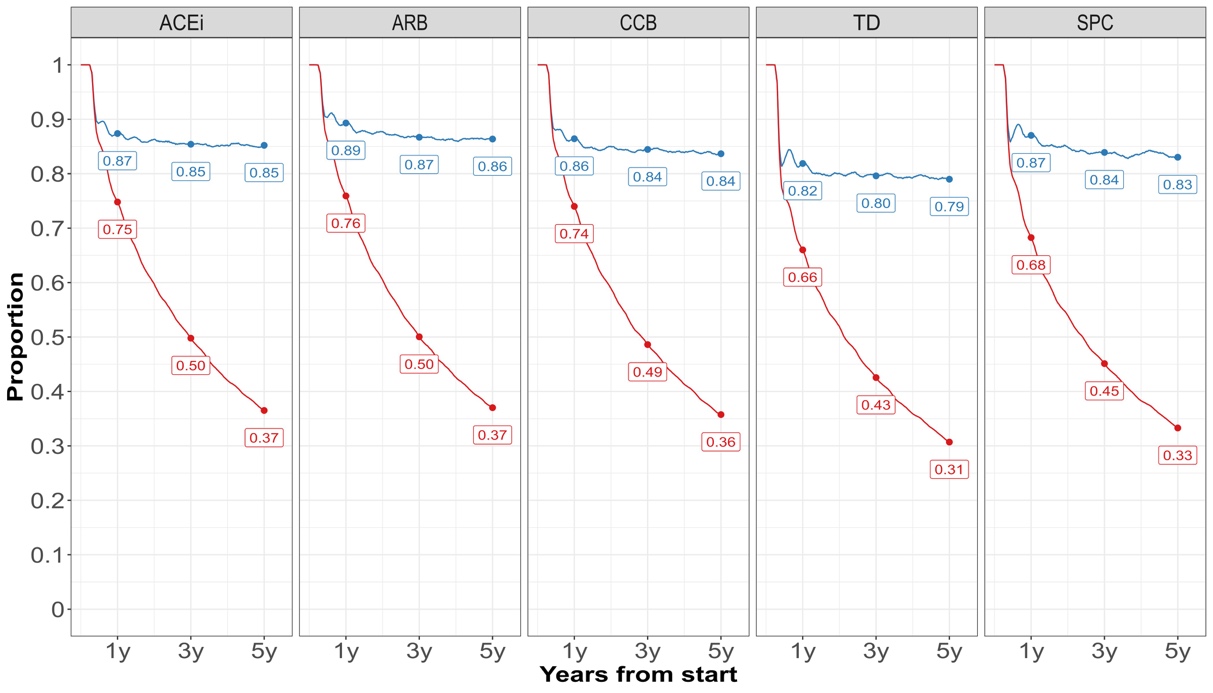


The red line illustrates continuous persistence and the blue line displays point persistence.

## Class persistence when changing to SPC containing original drug class ≠ class persistent

The model is set to median for continuous variables and to the most common level for categorical variables i.e time of initiation (2014-01-01), age (61 years), birth country (Sweden), highest education (high school), marital status (partner), total income (290 000), obesity (no) and sex (male)

In an earlier version of the method, a person continuing their original drug class, but changing from monotherapy to a single pill combination (containing the original class) was not considered class persistent since the ATC code changed. This was later changed to the method described in the article. For transparency the results of the earlier method can be seen in Figure S29.

### Figure S28. Class persistence. SPC ≠ class persistent


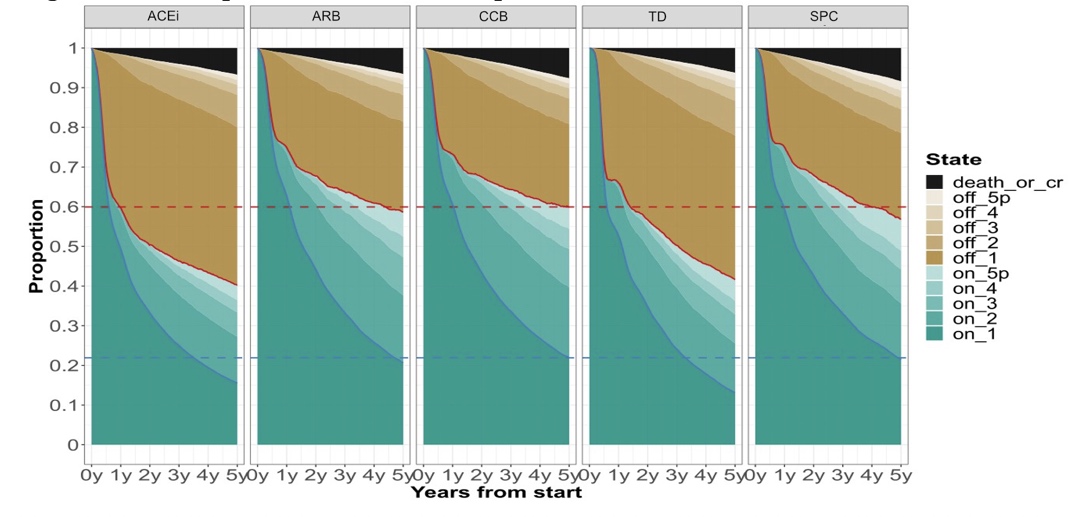

Supplement: Supplementary Material [file mmc1.docx]
